# Medical school admission processes to target rural applicants: an international scoping review and mapping of Australian practices

**DOI:** 10.1186/s12909-025-07234-3

**Published:** 2025-05-06

**Authors:** Jordan Fox, Jessica Beattie, Janelle McGrail, Diann Eley, Lara Fuller, Wendy Hu, Catherine Keniry, Srinivas Kondalsamy Chennakesavan, Lyndal Parker-Newlyn, Katharine Reid, Lucie Walters, Matthew McGrail

**Affiliations:** 1 The University of South Australia, PO Box 4143, Rockhampton, QLD 4700 Australia; 2 Rural General Practice (Program Development and Support), Deakin University, Warrnambool, Vic Australia; 3 The University of Queensland Rural Clinical School, Rockhampton, QLD Australia; 4 The University of Queensland, Brisbane, QLD Australia; 5 Rural Community Clinical School, Deakin University, Geelong, Vic Australia; 6 Western Sydney University, Sydney, NSW Australia; 7 Charles Sturt University School of Rural Medicine, Orange, NSW Australia; 8 The University of Queensland Rural Clinical School, Toowoomba, QLD Australia; 9 University of Wollongong, Wollongong, NSW Australia; 10 University of Melbourne, Melbourne, VIC Australia; 11 The University of Adelaide, Mount Gambier, SA Australia

**Keywords:** Social accountability, Social missions, Medical education, Admission and selection, Medical workforce

## Abstract

**Background:**

Recruiting more rural origin applicants to medical school is a high priority for addressing rural workforce shortages, given evidence demonstrating they are more likely to practice rurally. Whether strategies used to select rural applicants for Australian medical schools are evidence-based is yet to be evaluated in depth. The primary aim of this study was to identify admission and selection processes utilised by Australian medical schools to target rural applicants and evaluate whether they are evidence-based against international literature. A secondary aim was to document the volume and type of publicly available information regarding admission and selection processes in Australian medical schools.

**Methods:**

The study comprised two stages: (1) identified and summarised admission elements used in each Australian medical school (all pathways), how they are weighted and used to select applicants, and what strategies are used to specifically target rural origin applicants, as determined by publicly available information; and (2) conducted a scoping review of international literature to document the evidence relating to medical school admissions processes which target rural applicants and how they impact the selection of students.

**Results:**

Australian medical schools employ a combination of academic performance and standardised tests to rank applicants for interview and admission; however, there was extensive variation in the specific admission elements assessed and how they were weighted and used to rank applicants. All except one medical school tailored entry requirements for rural applicants. Scoping review data revealed that on average, rural applicants achieve lower scores on academic performance metrics, although this difference was less pronounced in graduate entry compared to school leaver pathways. Overall, rural applicants achieved lower scores on standardised tests and results relating to interview performance were inconsistent across studies.

**Conclusions:**

In addressing the lower mean scores from rural applicants across commonly used admission tests, our evidence confirms that modified admission processes for this applicant group are needed and are being implemented across Australian medical programs. Medical schools should continue to implement rurally-targeted admissions strategies such as adjusted entry requirements, quotas, and alternative entry pathways while setting clearly defined selection targets and evaluating the effectiveness of any strategies.

**Supplementary Information:**

The online version contains supplementary material available at 10.1186/s12909-025-07234-3.

## Introduction

Medical school admission is a highly competitive process, attracting a large proportion of applicants who rank in the highest decile of all tertiary applicants, but only a small proportion can be offered a place in each program. Medical schools implement rigorous admission and selection processes comprising of multiple elements and phases, seeking both high-level cognitive abilities and interpersonal skills required by future doctors. They have a responsibility to ensure equity (fairness) within the admissions process, whilst also needing to select applicants who are most likely to be well- suited to the job and become highly competent doctors [1]. A further consideration of growing relevance is ensuring diversity of selected applicants who will meet the health needs of the communities in which they are trained (i.e., social accountability), which requires that underrepresented groups are not disadvantaged during the selection processes [2–4].

Medical schools utilise admission processes and strategies which ideally will support achieving the goals or mission of their program, including those related to social accountability. The quality of implementation varies, with limited available evidence as to whether these strategies are effective in helping to increase, or even overrepresent, the proportion of successful applicants from underrepresented groups, such as rural applicants. A key finding of Ellaway et al.’s. 2018 scoping review, which explored associations between medical school’s social missions and their admissions processes, was that evidence was lacking into whether these socially accountable admissions approaches were effective [4]. In addition, while the review was internationally focussed, the majority of studies were from the United States (US). A 2020 review by Soemantri et al., also explored medical school admissions, focussing on underrepresented groups, but was limited to seven Asian countries [5]. Many geographical regions have not been evaluated in detail relating to whether admission and selection processes align with community health needs.

Another important aspect of equitable and socially accountable admission processes is whether potential applicants can easily identify and access relevant information. Many governing bodies, such as the Australian Medical Council (AMC) require that admissions information is publicly available as a requirement of their accreditation [6]. Transparency is particularly important for disadvantaged and underrepresented applicants [7] as they often rely on free and publicly available resources to source admissions information. Therefore, when evaluating the effectiveness of socially accountable admissions processes, the transparency and accessibility of information should be considered.

Most medical schools assess applicants on their academic (cognitive) ability and some personal attributes (non-cognitive). Scoring highly on both domains is considered desirable for commencing medical students; however, there is no consensus on how the various elements should be measured and weighted during admission to medical school, particularly with respect to meeting selection diversity and social accountability targets. Cognitive abilities are generally quantified via academic performance from high school (school leaver entry pathway) or recently completed university study (graduate entry pathway), and by standardised aptitude and personality tests (henceforth referred to as standardised tests) designed for applicants for medical and health professional entry courses. In Australia, most applicants complete either the University Clinical Aptitude Test (UCAT-ANZ) [8] or Graduate Medical School Admissions Test (GAMSAT) [9]. For both academic performance [10, 11] and standardised selection tests [12–14], rural origin cohorts tend to have significantly lower mean scores when compared to non-rural applicants [15].

Similar performance trends have been reported when non-cognitive attributes are assessed at medical school admission, such as through interviews, with lower scores reported for rural compared to non-rural applicants. Panel interviews or Multiple Mini-Interviews (MMI) are frequently used to assess non-cognitive attributes, with the MMI gaining global popularity over the last 10 years. Due to its multiple stations, assessors, and perspectives of the candidate, the MMI is often believed to offer superior validity and reliability compared to a panel interview [16]. However, studies directly comparing panel interview and MMI formats have suggested that the lower reliability of panel interviews could relate to biases such as training of interviewers and standardisation of processes, rather than limitations inherent to their format [17]. Furthermore, the use of MMIs does not necessarily equate to greater diversity in selection, as MMIs may similarly disadvantage rural applicants [18].

Lower scores on commonly used selection measures by rural origin medical school applicants have led to the adoption of additional strategies to recruit suitable rural applicants. This is particularly common for training pathways with a rural focus, which aim to fill most places with candidates who demonstrate a clear rural interest/intent, including those of childhood rural origin who are more likely to practice rurally. These strategies may include quotas, adjustment points, screening of rural origin/interest at interview, and in some cases, an essay or personal statement. It remains unclear which of these strategies are most effective and whether they do enough to counteract the systemic disadvantages experienced by rural applicants. Available evidence indicates that rural applicants admitted on lower entry scores perform similarly within medical programs to those admitted on higher scores [12, 19]. Therefore, evidence to date suggests that medical schools can implement socially accountable admissions processes without compromising the quality of the program or its graduates.

Australia has heavily invested in geographically dispersing its medical school training, with significant recent growth in the number of medical programs with extended rural training pathways. Building on the Rural Health Multidisciplinary Training (RHMT) program, which includes a national distribution of Rural Clinical Schools, the 2018 ‘Stronger Rural Health Strategy’ funding introduced the Murray Darling Medical Schools Network, encompassing five new medical programs to be delivered in wholly rural locations, designed to help address workforce shortages in rural New South Wales and Victoria [20]. In late 2023, six additional ‘end-to-end’ rural medical programs were announced across Australia [21]. Considering this growth, it is important to evaluate admission processes across Australia’s medical school programs and rural pathways, including whether they are evidence-based, and how well they assist in meeting selection targets for rural origin applicants.

The aim of this study was to identify admission and selection processes utilised in Australian medical schools to target rural applicants and to evaluate whether they are evidence-based, as determined via consensus within international literature. A secondary aim was to determine the volume and type of publicly available information outlining admission and selection processes in Australian medical schools. To achieve these aims, two stages were used: (1) identify and summarise admission elements used in each Australian medical school (all pathways), how they are weighted and used to select applicants, and what strategies are used to specifically target rural origin applicants, as determined by publicly available information; and (2) conduct a scoping review of international literature to document the evidence relating to medical school admissions processes and how they impact the selection of applicants most likely to work rurally.

## Methods

The protocol for both stages of this study was registered on the Open Science Framework [22] prior to study commencement [23]. For the purpose of this study, ‘rural’ was considered as any non-metropolitan location, as determined by the medical school (stage one) or authors of the included studies (stage two). In an Australian context, rurality is typically classified using the Australian Statistical Geographical Classification-Remoteness Area or Modified Monash Model, which similarly categorise locations as metropolitan or non-metropolitan.

### Stage one

For stage one (mapping of admission practices), publicly available data were reviewed for each medical program (or pathway) that is delivered by one of the accredited medical schools on the AMC list [24]. This AMC list provides links to the official webpage of each medical school which was used as the primary source of information, alongside each jurisdiction’s tertiary admissions centre website and the Graduate Entry Medical School Admissions System (GEMSAS) website [25]. These websites host information provided by/endorsed by each medical school, rather than relying on unofficial sources. The search was conducted between December 2023 and February 2024.

Data extraction was completed using a template approved by all authors prior to registering the protocol. The template was piloted for two medical programs (all pathways) before minor refinements were made. For each program, data were extracted by two authors independently (JF and JM or JB) before data were compared and discussed to establish consensus. For all extracted pieces of information, the source website was recorded and screenshots were taken. For joint medical programs, data were separately extracted from the website of each medical school to determine whether the information was consistent, except where the admissions information was only provided on a single medical school’s website.

### Stage two

The scoping review was conducted and reported in accordance with the JBI Manual for Evidence Synthesis [26] and the Preferred Reporting Items for Systematic Reviews and Meta-Analyses Extension for Scoping Reviews (PRISMA-ScR) [27] (Supplementary Table 1). The draft search strategy developed as part of the protocol was revised after stage one to ensure completeness in keywords used. All authors approved the final search strategy.

Four electronic databases (PubMed, SCOPUS, EMBASE and Web of Science) were utilised. Search terms were drawn from three topic areas: medical school, admission/selection elements/processes, and rural origin/interest or social accountability. Each group of keywords were originally searched, separated by the Boolean operator ‘OR’ before the search strings were combined with ‘AND’. Truncation and wildcard searching were used and limits/filters were applied to narrow the results based on year of publication and article type. Keywords used to formulate the search and the complete search strategy for each database are provided as supplementary Tables 2 and 3, respectively.

#### Study selection and data extraction

The search was conducted on 1 May 2024. Search results from each electronic database were imported into Covidence software (Covidence systematic review software, Veritas Health Innovation, Melbourne, Australia) which facilitated de-duplication, screening, and data extraction. Articles were screened based on the inclusion and exclusion criteria outlined in Table 1. Prior to each screening stage (title/abstract and full-text), a selection of articles were screened by two authors (JF and JB) together to ensure consistency. All titles and abstracts were then screened independently by two authors (JF and JB) with any conflicts discussed to establish agreement before full-texts were then screened via the same process. For the extraction of quantitative and qualitative data, a template was designed in Covidence software, with all data extracted by the lead author (JF).

**Table 1:** Inclusion and exclusion criteria for stage two (scoping review)

	Inclusion criteria	Exclusion criteria
**Medical school**	• School leaver medical programs • Graduate entry (following university study) medical programs • Provisional entry (Entry to medical school is granted prior to the undergraduate degree, pending satisfactory academic performance) and lateral entry (students apply during their undergraduate degree for provisional entry to medical school) medical programs	• Other health programs (e.g., nursing, allied health, dentistry) • Additional training completed after medical school (e.g., specialty/vocational training)
**Admission/selection elements and associated processes**	• Any admission elements or processes used to screen or select applicants at any stage of admissions • Assessment of cognitive or non-cognitive outcomes used at any stage of admissions	• Any other elements or processes not specified in the inclusion criteria
**Rural origin/rural interest/social accountability**	• Studies reporting data from only rural applicants, as determined by the medical school or government definition of rural origin • Studies reporting comparative data from rural and metropolitan applicants, as determined by the medical school or government definition of rural origin • Studies reporting data relating to rural interest or openness to rural practice, measured at admission	• Studies reporting data from metropolitan applicants only • Studies reporting aggregated data from rural and metropolitan applicants • Studies reporting data relating to rural interest or openness to rural practice measured after being admitted to the medical program • Studies reporting data relating to social accountability but without an explicit rural focus
**Other**	• Quantitative, qualitative, or mixed methods study designs • Published on or after 01/01/2003 • Published in English • Original research (including theses)	• Reviews or meta-analyses • Editorials and commentaries • Conference abstracts/papers • Research protocols • Non-peer-reviewed sources

#### Data synthesis

A narrative summary of results and key findings is provided for stages one and two separately. The integration of stages one and two (comparing international evidence with Australian practice) is presented within the discussion section; given the broad range of outcomes investigated and data obtained, it was not feasible to formally compare stages one and two.

## Results

### Stage one– Mapping of admission practices

A summary of each admission pathway for all Australian medical programs is provided in Table 2, with details of admission requirements and processes for each pathway provided in Supplementary Table 4. Each program (*N* = 20; 18 medical schools, and two joint medical programs) reported between two and 12 different pathways into medicine, including a combination of school leaver, provisional entry, lateral entry and graduate entry pathways. Unless otherwise stated, counts reported below represent strategies used for the majority of the medical school’s pathway, noting that many used different processes and selection elements for special entry pathways such as for Indigenous applicants (see Supplementary Table 4).

**Table 2:** Overview of each medical training pathway in Australia

Medical school	Pathway	Course	Location and rural exposure	Entry type	Total and rural places in each pathway (if applicable)
The University of Adelaide	Domestic– Recent secondary education	Medical Studies/MD	Metropolitan campus Four-week to year-long rural placements	School leaver	136 domestic places (40 rural)
	Domestic– Higher Education Study	Medical Studies/MD	Metropolitan campus Four-week to year-long rural placements	School leaver	136 domestic places (40 rural)
	International	Medical Studies/MD	Metropolitan campus Four-week to year-long rural placements	School leaver	30
	Aboriginal Access Scheme	Medical Studies/MD	Metropolitan campus Four-week to year-long rural placements	School leaver	4
Australian National University	Health Sciences	MChD	Metropolitan campus One-week to year-long rural placements	Graduate	30 domestic (10 rural) and 20 international
	Philosophy (Honours)	MChD	Metropolitan campus One-week to year-long rural placements	Graduate	10 domestic and 10 international
	Domestic	MChD	Metropolitan campus One-week to year-long rural placements	Graduate	90 (29% rural)
	International	MChD	Metropolitan campus One-week to year-long rural placements	Graduate	20
	Aboriginal and Torres Strait Islander Pathway	MChD	Metropolitan campus One-week to year-long rural placements	Graduate	Not reported
Bond University	Undergraduate	Medical Studies/MD	Metropolitan campus	School leaver	144
	Graduate	Medical Studies/MD	Metropolitan campus	Graduate	36
	Lateral	Medical Studies/MD	Metropolitan campus	Lateral	Based on availability
Charles Sturt University (CSU) and Western Sydney University (WSU)	CSU - Domestic	MD	Rural campus	School leaver	Not reported
	CSU– Rural	MD	Rural campus	School leaver	Not reported (80% rural; all domestic pathways)
	CSU– First Nations	MD	Rural campus	School leaver	Not reported
	WSU - Domestic	MD	Metropolitan campus Rural placements available	School leaver	120 domestic places (total)
	WSU– Rural	MD	Metropolitan campus Rural placements up to one-year available	School leaver	15
	WSU– Aboriginal and Torres Strait Islander	MD	Metropolitan campus Rural placements up to one-year available	School leaver	Not reported
	WSU - International	MD	Metropolitan campus	School leaver	20
Curtin University	School leaver	MBBS	Metropolitan campus Opportunity for rural placements	School leaver	110 (25% rural) across all domestic pathways
	Non-school leaver course switchers	MBBS	Metropolitan campus Opportunity for rural placements	School leaver	110 (25% rural) across all domestic pathways
	Graduates	MBBS	Metropolitan campus Opportunity for rural placements	School leaver	110 (25% rural) across all domestic pathways
	Aboriginal and Torres Strait Islander	MBBS	Metropolitan campus Opportunity for rural placements	School leaver	110 (25% rural) across all domestic pathways
	International	MBBS	Metropolitan campus Opportunity for rural placements	School leaver	10
Deakin University	General domestic	MD	Metropolitan campus Rural placements available (years 3 and 4)	Graduate	136 across all pathways (25% rural + 30 places in the rural pathway)
	Rural	MD	Rural campus	Graduate	30
	International	MD	Metropolitan campus Rural placements available (years 3 and 4)	Graduate	15
	Indigenous entry	MD	Metropolitan campus Rural placements available (years 3 and 4)	Graduate	6
Flinders University	Undergraduate entry South Australia	Clinical Sciences/MD	Metropolitan campus	School leaver	Not reported
	Provisional entry Northern Territory	Clinical sciences/MD	Rural campus	Provisional	12
	Graduate entry South Australia	MD	Metropolitan campus Year-long rural placements available	Graduate	90 (28% total places rural)
	Graduate entry Northern Territory	MD	Rural campus	Graduate	18
	Aboriginal and Torres Strait Islander	MD	Rural or metropolitan campus	Graduate	Not reported
Griffith University	Domestic	MD	Metropolitan campus Rural placements available (years 3 and 4)	Graduate	198 across all pathways (proportion of places for rural applicants)
	International	MD	Metropolitan campus	Graduate	35
	First peoples health	MD	Metropolitan campus	Graduate	Not reported
James Cook University	Domestic	MBBS	Rural campus	School leaver	170
	International	MBBS	Rural campus	School leaver	Not reported
Macquarie University	General	MD	Metropolitan campus	Graduate	70
	International	MD	Metropolitan campus	Graduate	20
	Indigenous Pathway	MD	Metropolitan campus	Graduate	Not reported
The University of Melbourne	Standard (domestic/International)	MD	Metropolitan campus Most of the MD completed in clinical schools (including rural)	Graduate	355 across all pathways (30% rural)
	Guaranteed	MD	Metropolitan campus Most of the MD completed in clinical schools (including rural)	Graduate	Not reported
	Rural	MD	Rural campus	Graduate	30
	Indigenous	MD	Metropolitan campus Most of the MD completed in clinical schools (including rural)	Graduate	Not reported
Monash University	School leaver	Medical science/MD	Metropolitan campus Rural placements available for years 3–5	School leaver	~ 234 direct entry
	Graduate domestic	MD	Rural campus	Graduate	73 (30 rural)
	Graduate international	MD	Metropolitan campus	Graduate	Not reported
University of Newcastle and the University of New England	General domestic	Medical science/MD	Rural or metropolitan campus Rural placements available	School leaver	170 (30–33% rural)
	International	Medical science/MD	Metropolitan campus	School leaver	Not reported
	Equity pathway	Medical science/MD	Rural or metropolitan campus	School leaver	6
	Aboriginal and Torres Strait Islander	Medical science/MD	Rural or metropolitan campus	School leaver	Not reported
University of Notre Dame	General Freemantle	MD	Rural campus	Graduate	100 (28% of CSPs rural) across all pathways
	General Sydney	MD	Metropolitan campus Rural clinical school sites	Graduate	100 (28% of CSPs rural) across all pathways
	International Freemantle	MD	Rural campus	Graduate	10
	International Sydney	MD	Metropolitan campus Rural clinical school sites	Graduate	50
	Aboriginal entry - Freemantle	MD	Rural campus	Graduate	10
	Aboriginal entry - Sydney	MD	Metropolitan campus	Graduate	Not reported
	Priority pathway	MD	Rural or Metropolitan campus Rural clinical school sites	Graduate	Not reported
	Assured pathway	MD	Metropolitan campus Rural clinical school sites	Provisional	30
The University of Sydney	Undergraduate	Bachelor of Arts or Science/MD	Metropolitan campus	School leaver	30
	Graduate metropolitan	MD	Metropolitan campus	Graduate	300 places across all pathways
	Graduate rural	MD	Rural campus	Graduate	24
	Graduate– Indigenous Facilitated Entry	MD	Rural or metropolitan campus	Graduate	Not reported
	Indigenous Entry Scheme	MD	Metropolitan campus	Graduate	70
The University of New South Wales	Domestic	Bachelor of arts or medical studies/MD	Metropolitan campus	School leaver	198 across all pathways
	International	Bachelor of arts or medical studies/MD	Metropolitan campus	School leaver	100
	Rural	Bachelor of arts or medical studies/MD	Metropolitan campus year 1; remaining time rural	School leaver	198 across all pathways
	Gateway	Bachelor of arts or medical studies/MD	Metropolitan campus year 1; remaining time rural or metropolitan	School leaver	10
	Lateral	Bachelor of arts or medical studies/MD	Metropolitan campus	Lateral	10
	Aboriginal and Torres Strait Islander	Bachelor of arts or medical studies/MD	Metropolitan campus	School leaver	Not reported
The University of Queensland	Provisional - Brisbane	Any undergraduate/MD	Metropolitan campus	Provisional	135 (28% rural) across all pathways
	Provisional– Darling-Downs/SouthWest	Biomedical Science/MD	Rural campus	Provisional	135 (28% rural) across all pathways
	Provisional– Central Queensland/Wide Bay	Medical Sciences/MD	Rural campus	Provisional	135 (28% rural) across all pathways
	Provisional - Aboriginal and Torres Strait Islander -Brisbane	Any undergraduate/MD	Metropolitan campus	Provisional	Not reported
	Provisional - Aboriginal and Torres Strait Islander -Central Queensland/Wide Bay	Medical Sciences/MD	Rural campus	Provisional	Not reported
	Provisional - International	Any undergraduate/MD	Metropolitan campus	Provisional	195, all pathways (including Ochsner)
	Graduate - Brisbane	MD	Metropolitan campus	Graduate	135 (28% rural across all pathways)
	Graduate– Darling-Downs/SouthWest	MD	Metropolitan campus (year 1), rural years 2–4	Graduate	30 (28% rural across all pathways)
	Graduate– Central Queensland/Wide Bay	MD	Metropolitan campus (year 1), rural years 2–4	Graduate	60 (28% rural across all pathways)
	Graduate - International	MD	Metropolitan campus	Graduate	Not reported
	Graduate - Ochsner	MD	Metropolitan and international	Graduate	Not reported
	Graduate- Aboriginal and Torres Strait Islander	MD	Not reported	Graduate entry	Not reported
The University of Tasmania	Domestic	Medical sciences/MD	Rural campus	School leaver	50% rural across all domestic pathways
	Rural	Medical sciences/MD	Rural campus	School leaver	50% rural across all domestic pathways
	International	Medical sciences/MD	Rural campus	School leaver	NA
	Aboriginal and Torres Strait Islander	Medical sciences/MD	Rural campus	School leaver	Not reported
The University of Western Australia	Domestic - School leaver	Biomedicine/MD	Metropolitan campus Year-long rural placements available	Provisional	103 (30 rural)
	International - School leaver	Biomedicine/MD	Metropolitan campus	Provisional	40 (0 rural)
	Indigenous - School leaver	Biomedicine/MD	Metropolitan campus Year-long rural placements available	Provisional	10% of available places
	Graduate - Domestic	Biomedicine/MD	Metropolitan campus Year-long rural placements available	Graduate	103 (30% rural)
	Graduate - International	Biomedicine/MD	Metropolitan campus	Graduate	20
	Graduate - Indigenous	Biomedicine/MD	Metropolitan campus Year-long rural placements available	Graduate	10% of available places
The University of Wollongong	General	MD	Rural campus	Graduate	84 across all pathways
	Rural	MD	Rural campus	Graduate	39
	International	MD	Rural campus	Graduate	15
	Aboriginal and Torres Strait Islander	MD	Rural campus	Graduate	4

*Note*: MD = Doctor of Medicine; MChD = Medicinae ac Chirurgiae/Doctor of Medicine and Surgery; MBBS = Bachelor of Medicine, Bachelor of Surgery

Of the 20 programs, 18 reported a separate pathway for Indigenous applicants, and seven have a separate pathway for rural applicants, noting that all other medical schools except one report one or more adjusted entry requirements for rural applicants. Of the 18 programs using interviews as part of selection, applicants are shortlisted for interview based on standardised tests (*N* = 4), standardised tests and academic performance (*N* = 4), standardised tests, academic performance and bonus points/adjustments (*N* = 2; the type of bonus points/adjustments and how these are allocated vary between medical schools), or standardised tests and a portfolio (*N* = 1). A further five medical schools use different processes for school leaver and graduate entry pathways, with three of these using standardised tests only for school leaver entry pathways and standardised tests and academic performance for graduate entry. Lastly, two medical schools use different processes for general and rural pathway applicants with one omitting the requirement for standardised tests for all rural applicants and one omitting this for only local rural applicants. When shortlisting for interview, standardised tests are weighted at 50–100% (two programs used two tests weighted 30–35% each), academic performance at 35–50%, the portfolio at 50%, and bonus points/adjustments at 10% (or not reported). One university uses staged rankings of academic performance and then standardised testing before offering interviews and four programs did not report the weightings of at least one admissions element used to shortlist applicants for interview.

When making admission offers, programs utilise many different elements and combinations. This includes standardised tests, academic performance and interview (*N* = 6), standardised tests and interview (*N* = 2), standardised tests and academic performance (*N* = 2), academic performance and interview (*N* = 1), interview only (*N* = 1), online application, academic performance, rurality/Indigenous background and interview (*N* = 1), standardised tests, academic performance, interview and bonus points (*N* = 1), or standardised tests, interview and a portfolio (*N* = 1). A further four programs used different processes for general and rural applicants by omitting the requirement for standardised tests for all rural (*N* = 1) or local rural applicants along with including a written statement (*N* = 1) and including a rural rating for rural pathway applicants (*N* = 2). Lastly, one program uses standardised tests only for school leaver entry. When making admissions offers, academic results are weighted at 25–90%, standardised tests at 10–33%, interview performance at 33–100%, written application at 25%, portfolio at 50%, and rural rating at 50%. Two programs reported the weightings contributed by multiple admission elements but not whether they were equally weighted, and four programs did not report weightings used for any admission elements when making offers.

Academic performance for school leaver and provisional entry programs predominantly used their Australian Tertiary Admissions Rank (ATAR), a score of 0 to 99.95 representing a high school student’s ordered position relative to other students in their cohort [28]. Of the 12 programs which utilise ATAR, all prescribed minimum ATAR requirements and this ranged from 90.0 to 99.95 for general applicants and 90–95 for rural applicants, which sometimes included some adjustments for eligible rural applicants. Of the 15 programs with graduate entry pathways, the applicants Grade Point Average (GPA) was used, with 13 prescribing minimum requirements which range from 4.9 to 6.5 for general applicants and 4.5–6.5 on a 7-point scale for rural applicants. Standardised test performance was commonly assessed using either UCAT-ANZ for school leavers or GAMSAT for graduate entry applicants. Of the 11 programs using UCAT-ANZ, one prescribed minimum requirements, with general and rural applicants required to be above the 50th percentile. Of the 11 programs using GAMSAT, all set minimum requirements with general applicants required to achieve 50–64 on each section and 50–76 overall and rural applicants required to achieve 50 on each section and 50–64 overall. Of the 18 programs using interviews, one required a minimum score of 50% for all applicants and one specified that applicants in the rural pathway must pass the interview.

Criteria used to determine rural origin was reported for 18 programs, with only one not making any adjustments for rural applicants. Either the Australian Statistical Geography Standard Remoteness Area (RA) [29] or Modified Monash Model (MMM) [30] classifications were used. Sixteen programs defined rural as five consecutive or 10 cumulative years spent in an RA2-5/MM2-7 region over any time period (*N* = 13), since age five (*N* = 1), since year 1 of primary school (*N* = 1), or between the ages of 5 and 18 (*N* = 1). Of these 16 programs, one classified MM1 (normally defining metropolitan) applicants in the same catchment as their regional-based program as ‘rural’, one did not classify applicants as rural if they completed year 12 at a metropolitan high school, and one only deemed applicants to be ‘rural’ if they were from the same state as the program. Other definitions without specific residency durations included ‘coming from’ an MM2-7 area (*N* = 1) and ‘living/studying in’ an MM2-7 area (*N* = 1). Six medical programs also applied one or more place-based quotas including a proportion of interviews from the same state and/or location as the program (*N* = 3), a proportion of places quarantined for applicants from the same state (*N* = 3), and an equal number of interview invitations for local, rural, and metropolitan applicants (*N* = 1).

Strategies used to prioritise rural applicants (applied by 19 of the 20 programs) included ATAR/GPA adjustments or reduced requirements (*N* = 10), separate rural pathway(s) (*N* = 7), sub-quotas (*N* = 5), different admission elements or weightings used for general and rural applicants (*N* = 3), GAMSAT adjustments or reduced requirements (*N* = 3), place-based tiers used to select for interview/offers (*N* = 2), and bonus points added to the interview and selection rank (*N* = 1), noting that many programs used multiple strategies and three did not clearly report how adjustments were made or how rural applicants are prioritised within the admissions processes. Of the 18 programs offering bonded medical places (whereby graduates from the program have a rural return of service obligation), places were allocated by offering non-bonded places to highest ranked applicants (*N* = 6), based on applicant ranking and preference (*N* = 3), using separate course codes for bonded and non-bonded places (*N* = 2), and all applicants in a specific pathway mandated bonded places (*N* = 2), with some programs using multiple strategies for allocating bonded places for different pathways, and seven programs not reporting how bonded places are allocated.

An overview of interview details for each pathway are provided in Table 3. Of the 18 programs routinely using interviews, these were primarily conducted online (*N* = 9) rather than face-to-face (*N* = 3), with one program interviewing local applicants face-to-face and others online, and five programs not reporting interview modality. Most programs use an MMI (*N* = 14), with one not reporting interview type and some programs using a panel interview for special entry pathways such as for Indigenous and international applicants. Often it was unclear whether applicants in all pathways within the same program undertook the same interview processes. The number of MMI stations reported ranged from 4 to 8 with duration ranging from 5 to 10 min per station. Assessment of rural attributes during interview was only reported for two individual pathways.

**Table 3:** Interview details for each Australian medical pathway

Medical school	Pathway	Interview type	Interview details	Rurally focussed interview questions reported?
The University of Adelaide	Domestic– Recent secondary education	MMI	Online Six x 10-minute stations	No
	Domestic– Higher Education Study	MMI	Online Six x 10-minute stations	No
	International	Not reported	Online 15 min	No
	Aboriginal Access Scheme	Panel	Online	No
Australian National University	Health Sciences	Not reported	Online	No
	Philosophy (Honours)	Not reported	Not reported	No
	Domestic	Not reported	Not reported	No
	International	Not reported	Online	No
	Aboriginal and Torres Strait Islander	Not reported	Not reported	No
Bond University	Undergraduate	MMI	Four stations 2–3 h inclusive of registration and debriefing	No
	Graduate	MMI	Four stations 2–3 h inclusive of registration and debriefing	No
	Lateral	MMI	Four stations 2–3 h inclusive of registration and debriefing	No
Charles Sturt University and Western Sydney Universiy	CSU - Domestic	MMI	Online Eight minutes per station Admissions questionnaire used to inform some interview questions	No
	CSU– Rural	MMI	Online Eight minutes per station Admissions questionnaire used to inform some interview questions	No
	CSU– First Nations	Panel	Questions about motivation, educational experience and aspirations, though the most important criterion is passion and desire to become a doctor	No
	WSU - Domestic	MMI	Online Eight minutes per station Admissions questionnaire used to inform some interview questions Assesses non-cognitive qualities important to patients and communities	No
	WSU– Rural	MMI	Online Eight minutes per station Admissions questionnaire used to inform some interview questions	No
	WSU– Aboriginal and Torres Strait Islander applicants	Panel	Questions about motivation, educational experience and aspirations, though the most important criterion is passion and desire to become a doctor Panel consists of professors of Aboriginal and Torres Strait Islander Health, an academic, and a community member	No
	WSU - International	Panel	Panel consists of staff members from the school of medicine	No
Curtin University	School leaver	MMI	Primarily face-to-face	No
	Non-school leaver course switchers	MMI	Primarily face-to-face	No
	Graduates	MMI	Primarily face-to-face	No
	Aboriginal and Torres Strait Islander	Two-day pre-admissions program	Assessed for key personal attributes necessary for success in the MBBS course through various engaging activities. Also gives applicants an understanding of learning and teaching at the university and supports available for students	No
	International applicants	MMI	In person (preferred) or online	No
Deakin University	General domestic	MMI	Six x five minute-stations	Yes
	Rural	MMI	Six x five minute-stations	Yes
	International	Panel	Not reported	No
	Indigenous entry	Not reported	Not reported	Not reported
Flinders University	Undergraduate South Australia	Unclear	Unclear	No
	Provisional Northern Territory	NA	NA	NA
	Graduate South Australia	Panel	In person for local residents otherwise online Assess qualities considered important both for success in the medical program and in subsequent medical practice 40–45 min	No
	Graduate Northern Territory	Panel	In person for local residents otherwise online Assess qualities considered important both for success in the medical program and in subsequent medical practice 40–45 min	No
	Aboriginal and Torres Strait Islander	Panel	In-person Financial assistance available to attend the interview Unclear if the same structure/format as the other interviews	No
Griffith University	Domestic	Resembles an MMI	Online 35 min for five scenarios	No
	International	Not reported	Not reported	No
	Aboriginal and Torres Strait Islander	Panel	Representatives from the student success unit, office of the Deputy Vice Chancellor, and medical school	No
James Cook University	Domestic	Panel	Online Identify attributes suited to a career in medicine The interview panel will consist of a medical practitioner, an academic staff member and a member of the community	No
	International	Not reported	Not reported	No
Macquarie University	General	MMI	Face-to-face	No
	International	MMI	Online Modified MMI	No
	Indigenous pathway	Not reported	Not reported	No
The University of Melbourne	Standard (Domestic/International)	MMI	Online Eight x five minute stations	No
	Guaranteed entry	MMI	Online Eight x five minute stations	No
	Rural	MMI	Not reported	Yes
	Indigenous pathway	MMI then panel	*MMI*: Online Eight x five minute stations *Panel*: Panel chaired by the Associate Dean (Indigenous) of the Faculty (or their nominee), and other appropriate Indigenous representatives in place of the GAMSAT	No
Monash University	School leaver	MMI	Online Six x 10 min stations	No
	Graduate domestic	MMI	Online Five x 10 min stations	No
	Graduate international	Not reported	Not reported	No
The University of Newcastle and The University of New England	General domestic	MSA	Eight x eight minute stations	No
	International	MSA	Not reported	No
	Equity pathway	MSA	Eight x eight minute stations	No
	Aboriginal and Torres Strait Islander	MSA	Eight x eight minute stations Where possible, panels include Aboriginal and Torres Strait Islander representatives from the medical school or community	No
The University of Notre Dame	General Freemantle	MMI	Online, recorded interview At least seven stations	No
	General Sydney	MMI	Online, recorded interview At least seven stations	No
	International Freemantle	Panel and MMI	Online, recorded interview	No
	International Sydney	Panel and MMI	Online, recorded interview	No
	Aboriginal entry Freemantle	Panel	Interviewed by members of the Aboriginal Health team	No
	Aboriginal entry Sydney	Not reported	Specific to the pathway	No
	Priority pathway	Not reported	Not reported	No
	Assured pathway	Not reported	Not reported	No
The University of Sydney	Undergraduate	Panel	Not reported	No
	Graduate metropolitan	No interview	NA	NA
	Graduate rural	No interview	NA	NA
	Graduate Indigenous Facilitated Entry	No interview	NA	NA
	Graduate international	No interview	NA	NA
The University of New South Wales	Domestic	Panel	Face to face Two interviewers who are academic staff, medical practitioners or community representatives	NA
	International	Panel	Online	NA
	Rural	Panel	Face to face Two interviewers who are academic staff, medical practitioners or community representatives	NA
	Gateway	Panel	Face to face Two interviewers who are academic staff, medical practitioners or community representatives	NA
	Lateral	Panel	Face to face Two interviewers who are academic staff, medical practitioners or community representatives	NA
	Indigenous Entry Scheme	NA	NA	NA
The University of Queensland	Provisional - Brisbane	MMI	Several stations	No
	Provisional– Darling-Downs/SouthWest	MMI	Eight stations x 10 min Identify candidates who are suitable to enter the pathway and eventually be a medical doctor in a rural, remote or regional setting.	Yes
	Provisional– Central Queensland/Wide Bay	MMI	Eight stations x 10 min	No
	Provisional - Aboriginal and Torres Strait Islander - Brisbane	Not reported	Not reported	No
	Provisional - Aboriginal and Torres Strait Islander– Central Queensland/Wide Bay	Panel and MMI	Semi-structured interview before a recommendation is made on whether the applicant should complete the MMI	No
	Provisional - international	MMI	Several stations	No
	Graduate - Brisbane	MMI	Several stations	No
	Graduate– Darling-Downs/SouthWest	MMI	Several stations	No
	Graduate– Central Queensland/Wide Bay	MMI	Several stations	No
	Graduate - international	MMI	Several stations	No
	Graduate - Ochsner	MMI	Several stations	No
	Graduate - Aboriginal and Torres Strait Islander	Not reported	Not reported	No
The University of Tasmania	Domestic	No interview	NA	NA
	Rural	No interview	NA	NA
	International	No interview	NA	NA
	Aboriginal and Torres Strait Islander	Panel	With representatives of the admissions committee	No
The University of Western Australia	School leaver - Domestic	MMI	Eight stations x seven minutes	No
	School leaver - International	Panel	50 min including registration Two interviewers	No
	School leaver - Indigenous	Two-day assessment Panel interview	Not reported	No
	Graduate - Domestic	MMI	Eight stations x seven minutes	No
	Graduate - International	MMI	Eight stations x seven minutes	No
	Graduate - Indigenous	Not reported	Unclear if different to school leaver entry	No
The University of Wollongong	General	MMI	Online	No
	Rural	MMI	Online	No
	International	MMI	Online	No
	Aboriginal and Torres Strait Islander	MMI and panel interview	Panel interview: Discuss understanding of Indigenous health and social issues; includes Aboriginal and Torres Strait Islander community members and university staff	No

*Note*: MMI = Multiple Mini-Interview; MSA = Multiple Skills Assessment; NA = Not Applicable

### Stage two– Scoping review

**Fig. 1:**
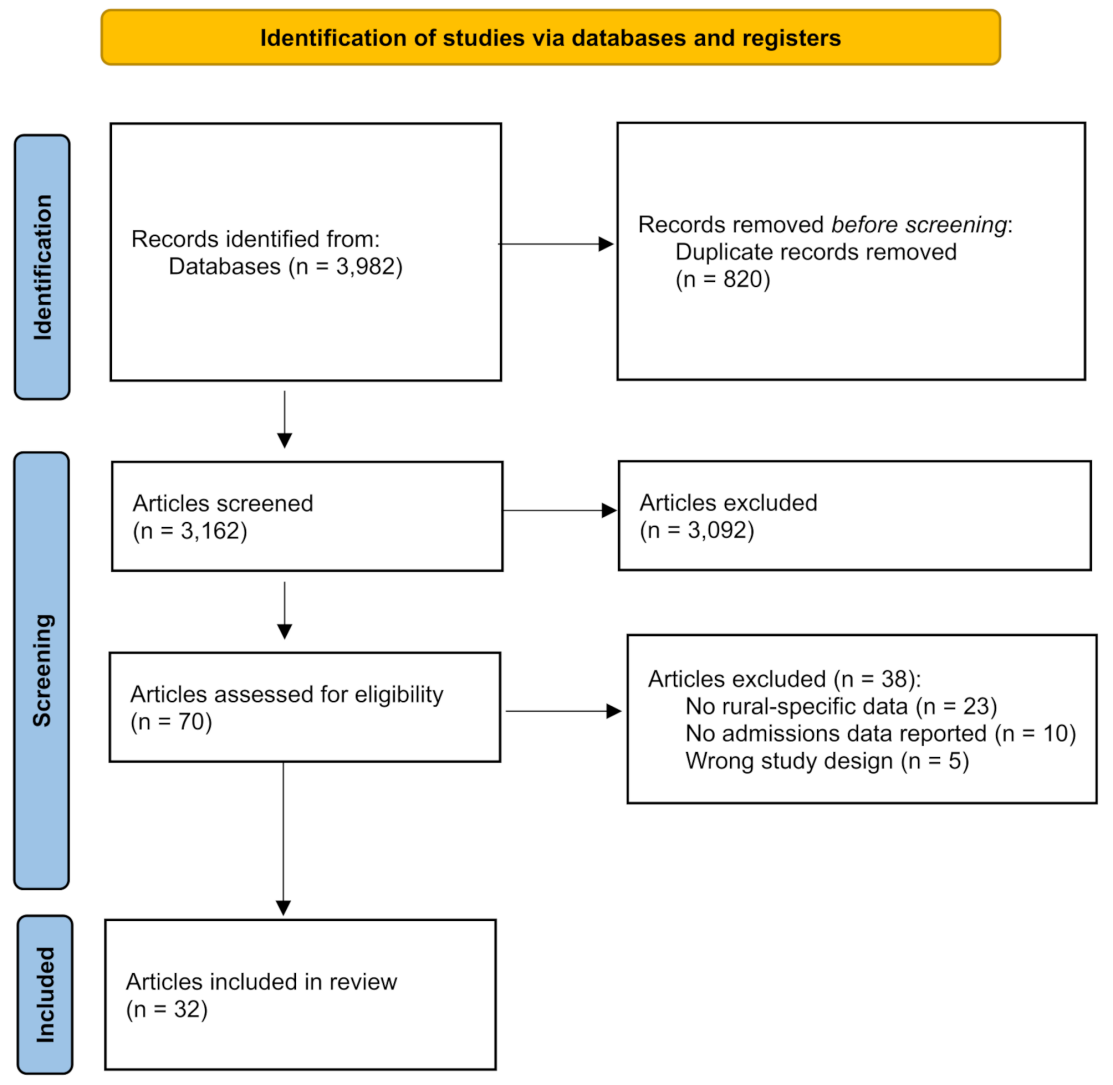
Flowchart of article screening and inclusion

The initial search yielded 3,982 results (PubMed: 1,683; SCOPUS: 1,136; EMBASE: 1,029; Web Of Science: 134) of which 820 were duplicates, leaving 3,162 records for title and abstract screening. After title and abstract screening, 70 full-text articles were assessed and of these, 38 articles were excluded leaving 32 articles for inclusion in the review (Fig. 1). An overview of the admissions processes investigated, aims, study designs, participants, and outcomes of interest for each included article is provided in Supplementary Tables 5 and quantitative and qualitative results from each included article are summarised in Supplementary Tables 6 and 7, respectively.

### Characteristics of included studies

Studies were conducted in Australia (*N* = 13; 41%), the US (*N* = 8; 25%), Canada (*N* = 4; 13%), Japan (*N* = 2; 6%), New Zealand (*N* = 1; 3%), Taiwan (*N* = 1; 3%), Malaysia (*N* = 1; 3%), Nepal (*N* = 1; 3%), and one was multi-national (3%). Studies were primarily quantitative (*N* = 26; 81%) and of these, 20 (77%) involved analysing administrative data, three (12%) utilised quantitative surveys, and three (12%) used a combination of quantitative surveys and administrative data. Of the other six studies, one conducted qualitative interviews, one conducted a mixed methods survey, and four were mixed-methods utilising multiple sources of data.

### Quantitative findings

Three Australian studies found that rural applicants had lower ATAR scores on average than non-rural applicants [19, 31, 32]. One also showed that Overall Position (OP) scores (used prior to ATAR) were progressively lower as the applicant’s rurality classification increased [33]. ATAR scores were also lower for applicants (rural and metropolitan) applying for the first time compared to applicants who were re-applying; however metropolitan applicants were more likely to re-apply if they were initially unsuccessful [34].

Regarding GPA, five studies from Australia and Canada reported that on average, rural and non-rural applicants had similar GPA scores [11, 35–38], one study from Australia reported lower GPA scores of rural applicants [12], and one study from the US reported higher GPA of rural applicants [39]. In addition, two studies (Australia and New Zealand) reported that GPA was lower for applicants admitted via the rural compared to the general entry pathway [10, 40].

When assessing standardised test scores used for selection in school leaver pathways, three Australian studies showed that on average, rural applicants scored lower on all Undergraduate Medical and Health Sciences Admissions Test (UMAT, which preceded the current UCAT-ANZ) sections and overall, compared to non-rural applicants [13, 19, 31] and one showed that rural applicants scores were lower for non-verbal communication and overall, but higher on understanding people [32]. UMAT scores were also lower for applicants entering via the rural compared to the general entry pathway [41]. In another study, UMAT scores in one section (non-verbal communication) progressively decreased as applicant’s rurality (remoteness) increased [42]. Furthermore, UMAT scores were higher for applicants re-applying compared to those applying for the first time and metropolitan applicants were more likely to re-apply [34]. On the UCAT-ANZ, rural applicants scored lower on all sections and overall [13]. Lastly, on the National Centre Test (Japan), rural pathway applicants achieved lower scores than those in the standard entry pathway [43].

In assessing standardised tests used for graduate entry selection, in US and Canadian medical schools, two studies found that on average, rural applicants had lower Medical College Admissions Test (MCAT) scores for some sections (physical sciences in both studies and biological science in one study) but similar scores for verbal reasoning [35, 39]. A later study (following changes to MCAT) also reported that rural applicants had lower scores on all sections except verbal reasoning [36]. Two studies showed that overall MCAT scores were lower for rural applicants [14, 36] and one showed similar MCAT scores for rural and non-rural applicants [38]. One Australian study reported similar GAMSAT scores in rural and metropolitan applicants [12] and another reported that applicants entering via the rural pathway had lower GAMSAT scores than those entering via the general pathway [10].

Regarding interviews, two studies from Australia and Malaysia reported similar MMI scores for rural and non-rural applicants [12, 44] and one Canadian study reported lower MMI scores for applicants graduating from rural high schools and applicants with rural connections [36]. Another study from Malaysia found that during MMIs, rural applicants scored lower on ethics and professionalism but had similar scores for motivation, teamwork, communication, and general impressions [44]. When evaluating an Australian MMI which included rural-specific questions, rural applicants were more likely to feel they had an advantage over other applicants [45]. For panel interviews, two Australian studies reported similar scores for rural and metropolitan applicants [32, 39] and two other Australian studies found that panel interview scores were lower for rural applicants [31, 33]. Two Australian studies also found that students entering via the rural pathway had lower panel interview scores [10, 41]. One Australian study did not report the interview format but found lower average scores for rural applicants [19] and a study from the US assessing both panel interviews and MMIs found that regardless of format, average interview scores were similar for applicants from rural and non-rural counties [46].

In data relating to admission offers, one US study showed that a similar proportion of rural and non-rural applicants were interviewed and subsequently admitted [47], another two (US and Japan) revealed that the likelihood of rural and non-rural origin applicants being admitted was similar [11, 48], one Japanese study showed that rural and non-rural applicants had similar academic standing at entry [49], and in a Canadian study, average reviewer scores of the overall application were similar for rural and non-rural applicants [38]. In the same Canadian study, a higher proportion of rural applicants were interviewed but the proportion of rural and non-rural applicants admitted was similar [38]. Furthermore, one US study showed that rural applicants were less likely to be offered a place [39] and another US study showed that even if adjustments for rural applicants were doubled (via additional points added to GPA based on the applicant’s undergraduate institution), the proportion of rural applicants interviewed remained considerably lower than non-rural applicants [35]. A further US study reported that out-of-state applicants were less likely to be accepted than local rural and non-rural applicants [50] and an Australian study found that non-rural applicants were more likely to re-apply after being unsuccessful and the applicants re-applying were more likely to be short-listed for interview [34].

One study broadly assessing rurally-aligned admissions processes, found that rurally located medical schools were more likely to have targeted rural admissions processes [51]. Recruitment strategies to boost rural applicant numbers included career counselling/exploration/mentoring, academic enhancement initiatives, admissions preparation activities, and articulation agreements (formal agreements between two institutions allowing transfer between specific programs) [51, 52]. Students were identified and prioritised during selection based on a range of factors including graduating from a rural high school, growing up in a rural community, volunteering in a rural community, previous employment in a rural community, a partner/spouse receptive to living in a rural community, graduating from a public university, positive rural exposure, interest in family medicine, having a non-continuous path from high school, and being from a group underrepresented in medicine [51, 52].

Selection strategies used by US medical schools at the time of application to prioritise rural applicants included asking additional questions within the application, targeted financial aid, modified cutoffs for various admission elements, and dedicated rural places [51, 52]. Selection strategies used by US medical schools at interviews and when making admission offers included having rural physicians as interviewers, preferential scoring, alternative admissions processes, separate rural interviews, and different interview questions [51, 52]. One multi-national study showed that at admission, students admitted to socially accountable medical schools via rurally-aligned admissions processes, were more likely to be from a rural location and have rural practice intent when compared to the national average [53]. A Canadian medical school reported that by developing a rural score (assessing rural community service, rural connections, and rural employment) which was used to adjust the applicant’s composite score of all admission elements, admission scores of rural applicants increased by 15%, leading to a 29–33% increase in the number of eligible rural applicants and 22.4% increase in admission offers [54]. In an Australian medical school, implementing a rurally aligned admissions process increased the proportion of rural applicants admitted from 4 to 12% to 20–22% per year [37] and Upadhyay et al. reported that after implementing revised admissions processes in a Nepalese medical school, the proportion of rural students admitted increased from 3% to 11–15% per year [55]. An Australian study showed that even after introducing alternative admissions processes (ranking applicants based on academic performance, interview, and aptitude test results rather than academic performance alone), the rural applicants had lower tertiary entrance scores [41].

### Qualitative findings

Qualitative data largely related to the general use of rurally aligned admissions processes, rather than a specific selection element. Motivations for targeted admissions strategies in the US included meeting the school’s social mission and training healthcare providers to meet community needs and workforce diversity [51]. Socially accountable recruitment and admissions processes were resource-intensive requiring funding, human resources, and support from institutional leadership [51]. Challenges included time and personnel required to recruit rural applicants, high tuition costs and limited scholarships making it difficult to attract appropriate applicants, and difficulties during interviews in discerning applicants with genuine rural interest [51]. Recommendations for targeted admissions included institutional support, outreach to potential rural applicants (including appropriate resourcing) and moving towards holistic review rather than focussing solely on academic merit [51].

In assessing selection strategies to target rural applicants, one Australian study which aggregated findings from nine graduate entry medical schools found that selection for interview used a combination of GPA and GAMSAT, while admission decisions were based on GPA, GAMSAT, and interview, with one also using a portfolio [56]. Only three programs reported assessing rurality during the interview, two included rurally focussed MMI stations, and one explored rurality extensively in interviews for the rural cohort [56]. Five programs used rural interviewers, with all commenting that this can be difficult to achieve [56]. Elsewhere (multi-national), strategies for rurally-aligned selection included quotas, selecting based on personal attributes, community involvement in the selection process, and attracting appropriate applicants by marketing the school’s social accountability mission [53].

In an Australian study assessing applicant perceptions of an MMI which included rurally-aligned questions, most applicants reported that these questions gave rural applicants an advantage over metropolitan applicants [45]. There were conflicting comments about whether this was fair, with some applicants reflecting that it counteracted other disadvantages faced by rural applicants [45].

## Discussion

The purpose of this study was to capture admission and selection processes used by Australian medical schools to target rural applicants, based on publicly available information, and to contrast current practice against international ‘best practice’ evidence. The findings highlight the extensive variation in admissions processes across medical schools and individual admission pathways including the admission elements used, how they are weighted/aggregated, and additional strategies utilised for rural selection. While almost all medical schools reported some level of adjustment/reduced requirements or alternative admissions pathway for rural applicants, the scoping review findings showed that often rural applicants had lower mean scores on the various cognitive and non-cognitive admissions elements utilised and were less likely to be offered a place in the program. Regarding transparency in admissions processes, there were several areas where information was difficult to identify such as whether interview procedures were standardised across all entry pathways and how bonded places were allocated. In a small number of cases, inconsistent information was reported by the same medical school across different sections of their website, making it difficult to confidently identify admissions processes within these pathways and programs.

When comparing current practice with available evidence, a number of key findings and areas of future research were identified. The majority of medical schools used some form of standardised test for entry into school leaver and graduate entry pathways; however, in the scoping review literature there was a clear trend for rural applicants to have lower test scores than their metropolitan counterparts [13, 14, 19, 31, 36]. There was also a tendency for applicants from more remote locations to have lower admission scores, although a considerable proportion of studies conducted a binary comparison of rural and metropolitan applicants’ admission scores. Some programs have omitted the requirement for standardised tests for rural applicants or pathways and notably, in the relevant programs, this omission was only applied to graduate entry rather than school leaver pathways. In an Australian context, continued use of these tests for school-leavers is likely because ATAR results (quantifying academic performance during high school) are typically not available at the time that applications to university are due, thus making it impractical to use this information when shortlisting applicants for interview. While these logistical considerations are highly relevant for medical schools, it may be useful to consider whether alternative strategies for shortlisting (e.g., utilising predicted ATAR or prior academic performance) could be implemented to replace or lower the emphasis on standardised tests early in the admissions processes for applicants to school leaver pathways, particularly for rural applicants facing increased disadvantage (such as those from smaller rural/remote communities).

All medical schools and pathways evaluated prior academic performance when ranking applicants for interview and/or admission. The scoping review revealed that on average, rural applicants had lower scores on academic metrics; however, performance differences between rural and metropolitan applicants were less substantial for metrics used for graduate entry selection (e.g., GPA) compared to those used for school leaver entry (e.g., ATAR). It may be that once at university, differences between rural and metropolitan applicants become less apparent, whereby they are exposed to the same environment and similar support structures; however, relying on this ignores the upstream barriers for rural applicants entering university. With the majority of medical schools implementing adjustments or lower academic requirements for rural applicants, particularly within school leaver pathways, it appears that Australian medical schools are acting in accordance with the available international evidence base, with respect to how they handle academic-based metrics during the admissions process whilst maintaining academic standards within the cohorts of medical graduates.

Regarding interviews, a combination of panel interviews and MMIs are utilised across Australian medical schools and pathways; however, in recent years MMIs have become considerably more common, despite variation in the format (e.g., in-person vs. online) as well as the number and duration of stations. When making offers of admission, there was also considerable variation in the weighting of interviews, ranging from ~ 30 to 100%, with some medical schools ranking applicants at multiple stages as admission decisions are made. The scoping review data revealed mixed results surrounding interviews with studies reporting either similar or lower scores for rural compared to metropolitan applicants. MMIs have been increasingly used as they are proposed as having better validity and reliability than panel interviews [16]. In spite of this, the data captured within this scoping review confirms past evidence that the use of MMIs does not necessarily support the selection of a more diverse student cohort, including those of rural origin [18]. Furthermore, in only a small proportion of pathways it was reported that the interview had a rural focus, although anecdotally this number is likely to be higher, despite not being explicitly reported. It is possible that incorporating a rural focus into interviews (e.g., capturing an applicant’s origin, understanding of rural practice, etc.) may help to both create equity during admissions and support medical schools in meeting their selection targets. Lastly, the composition of the interview panel/MMI examiners and what impact this has on student selection was not widely documented on university websites or within the scoping review literature. It is possible that the background of the interviewer, including whether they are of rural or metropolitan origin, influences how they relate to and subsequently score the applicant, and therefore, further research is needed.

Overall, international evidence indicates that irrespective of the admission elements assessed, there is a trend for rural applicants to receive lower scores. As such, these data imply that medical schools need to closely evaluate how these different elements are aggregated and weighted during admissions and consider whether the different elements are appropriate for selecting rural applicants or if alternative strategies are needed. In reviewing the international literature, it was often unclear whether adjustments led to meeting rural selection targets/mandates, even when studies reported on the impact of implementing rurally-aligned admissions processes. It is essential that strategies for providing rural applicants with equal opportunities to pursue medicine include both early intervention, such as high school engagement in rural and remote areas, as well as adjusted entry requirements. As such, we recommend that medical schools set clear admissions targets and plans for how these outcomes will be evaluated in working towards medical school cohorts which resemble the characteristics of the communities which they serve.

In regard to evaluating transparency in admissions information produced by Australian medical schools, we identified areas where information or clarity was lacking. For example, admissions information for each pathway was often detailed on separate documents or sections on the website while detailed information about the interview was only provided on one page (generally alongside information on the ‘general’ entry pathway). Because of this, it was often difficult to confirm whether interview processes were consistent across the different pathways. Therefore, medical schools should ensure consistency of relevant information across different areas of websites or more explicitly identify when interview formats are similar or different across entry pathways (e.g., whether applicants in all entry pathways complete a panel interview or MMI). Another aspect of admissions processes not widely reported was how bonded places were allocated. Without medical schools reporting processes for allocating bonded places, it remains unknown how the allocation of bonded places influences how medical school applicants prioritise their university preferences. Therefore, it is recommended that further information be made available regarding the allocation of bonded places and that research is conducted which explores how the allocation of bonded places influences medical school preferences and how likely rural and metropolitan applicants are to accept a place in a particular pathway or program.

### Strengths and limitations

This study comprehensively explored strategies used by Australian medical schools to target rural applicants. A strength is the combination of the two stages which allows current Australian practice to be compared against published international evidence. Furthermore, to maximise accurate collection of publicly available current admissions processes, all data were extracted by two authors per medical school independently and then compared. Utilising only publicly available data but without additional verification (such as interviewing admissions staff at each university) is a strength of the review as it provides valuable insights regarding how complete, accessible and transparent admissions information is, from an applicant perspective. Nevertheless, there are several limitations which need to be considered in the context of our findings. First, stage one (mapping of admission practices) reflects publicly available data captured at a single timepoint. Information reported on university websites may change regularly as admissions processes are updated and as additional data become available. Mapping data at a single timepoint also means that although the scoping review collated evidence from the last 20 years, the mapping of current practice does not reflect changes made to admissions processes previously and what impact these changes have had on the selection of rural applicants. A further limitation of the scoping review is that search terms were revised following stage one to ensure completeness. With stage one inclusive of Australian medical schools only, it is likely that the scoping review is therefore skewed towards Australian evidence and that some international literature and admission methods used outside of Australia may have been overlooked.

When interpreting these results, it should also be acknowledged that the term ‘standardised tests’ was used throughout but covers a range of assessments including but not limited to aptitude tests and personality assessments. When evaluating data relating to these tests/assessments, the specific instrument used along with its purpose and validity should be critically considered by both researchers and medical school representatives. Finally, this study’s focus was on increasing selection of rural applicants so scoping review data tended to include trends in average scores of rural and metropolitan medical school applicants. Although on average rural applicants had lower scores and admissions performance than metropolitan applicants, other demographic and personal characteristics such as socioeconomic status and ethnic or Indigenous background may co-exist and also warrant structures to be put in place to allow all potential applicants equal opportunities to pursue a career in medicine irrespective of their background.

## Conclusions

This study examined the breadth of current strategies used by Australian medical schools to target rural applicants and how this aligns with published international ‘best practice’ evidence. We also examined the volume and type of information publicly reported by Australian medical schools regarding their admissions processes across each of the different pathways, including relatively new rural-focused programs. Despite the importance of selecting medical school applicants who are likely to help address rural workforce shortages, available literature highlights that rural applicants have lower scores on average than their metropolitan counterparts across the range of admission elements used to assess cognitive and non-cognitive outcomes. As such, medical schools should continue to implement rurally-targeted admissions strategies such as adjusted entry requirements, quotas, and alternative entry pathways while setting clearly defined selection targets and evaluating the effectiveness of any strategies.

Engaging potential rural applicants well in advance of medical school applications (i.e., high school engagement) may also help to increase the pool of rural applicants considering medicine. Transparency in admission processes on official websites should also be prioritised as rural applicants often rely on these sources more heavily than metropolitan applicants.

## Electronic supplementary material

Below is the link to the electronic supplementary material.

Supplementary Material 1

Supplementary Material 2

Supplementary Material 3

Supplementary Material 4

Supplementary Material 5

Supplementary Material 6

Supplementary Material 7

## Data Availability

A summary of extracted data is provided as supplementary online material.

## Abbreviations

US: United States
AMC: Australian Medical Council
UCAT-ANZ: University Clinical Aptitude Test-Australia and New Zealand
GAMSAT: Graduate Medical School Admissions Test
MMI: Multiple Mini-Interview
RHMT: Rural Health Multidisciplinary Training
GEMSAS: Graduate Entry Medical School Admission and Selection Service
PRISMA-ScR: Preferred reporting Items for Systematic Reviews and Meta-Analyses Extension for Scoping Reviews
ATAR: Australian Tertiary Admissions Rank
GPA: Grade Point Average
RA: Australian Statistical Geographical Classification-Remoteness Area
MM: Modified Monash
OP: Overall Position
UMAT: Undergraduate Medicine and Health Sciences Admissions Test
MCAT: Medical College Admissions Test
