# Supplementary material for: Medical school admission processes to target rural applicants: an international scoping review and mapping of Australian practices
Source: BMC Med Educ. 2025 May 6;25:659. doi: 10.1186/s12909-025-07234-3 (PMC12057111; doi:10.1186/s12909-025-07234-3)
Supplement: Supplementary file 2 — Supplementary Material 2 [file 12909_2025_7234_MOESM2_ESM.docx]

**Supplementary Table 2.** Search terms and filters used to guide the search strategy for the scoping review

| **Input 1 (medical school):**  “medical schools [MeSH]”  “medical students [MeSH]”  “undergraduate medical education [MeSH]”  “undergraduate medical training [Title/Abstract]”  “Doctor of Medicine [Title/Abstract]”  “Bachelor of Medicine [Title/Abstract]”  “Bachelor of Surgery [Title/Abstract]”  “Doctor of Medicine and Surgery [Title/Abstract]” | **Limits:** English language, published on or after 01/01/2003, primary sources, subject (medicine), humans |
| --- | --- |
| AND |  |
| **Input 2 (admission/selection):**  “Applicant*[Title/Abstract]”  “Application* [Title/Abstract]”  “recruit* [Title/Abstract]”  “select* [Title/Abstract]”  “admit* [Title/Abstract]”  “admission* [Title/Abstract]”  “University Clinical Aptitude Test [Title/Abstract]”  “UCAT [Title/Abstract]”  “Undergraduate Medicine and Health Sciences Admission Test [Title/Abstract]”  “UMAT [Title/Abstract]”  “Multiple Mini-Interview* [Title/Abstract]”  “MMI [Title/Abstract]”  “Interview* [Title/Abstract]”  “Personal qualit* [Title/Abstract]”  “Grade Point Average* [Title/Abstract]”  “GPA [Title/Abstract]”  “Situational judgement [Title/Abstract]”  “Casper [Title/Abstract]”  “Quota* [Title/Abstract]”  “Graduate Medical School Admissions Test [Title/Abstract]”  “GAMSAT [Title/Abstract]”  “Australian Tertiary Admission Rank [Title/Abstract]”  “ATAR [Title/Abstract]”  “Overall Position [Title/Abstract]”  “OP [Title/Abstract]”  “Universities Admission Index [Title/Abstract]”  “UAI [Title/Abstract]”  “Tertiary entrance [Title/Abstract]”  “TER” [Title/Abstract]  “Psychometric test* [Title/Abstract]”  “Written statement* [Title/Abstract]”  “Written application* [Title/Abstract]”  “Personal statement* [Title/Abstract]”  “Subquota* [Title/Abstract]”  “sub quota* [Title/Abstract]”  “Bonus* [Title/Abstract]”  “Adjust* [Title/Abstract]”  “Score* [Title/Abstract]”  “Standardised test* [Title/Abstract]”  “Weighted Average Mark [Title/Abstract]”  “Weighted?Grade Point Average [Title/Abstract]”  “Weighted?GPA [Title/Abstract]”  “Medical Admissions Questionnaire [Title/Abstract]”  “MAQ [Title/Abstract]”  “Rank* [Title/Abstract]”  “Admissions Test* [Title/Abstract]”  “Weighted Mark [Title/Abstract]”  “Multiple Skills Assessment [Title/Abstract]”  “MSA [Title/Abstract]” |  |
| AND |  |
| **Input 3 (rural/regional/remote/social accountability):**  “social* accountab*[Title/Abstract]” AND (“regional* [all fields]” OR “rural* [all fields]” OR “remote*” [all fields])  “social* responsib*[Title/Abstract]” AND (“regional* [all fields]” OR “rural* [all fields]” OR “remote*” [all fields])  “social responsibilit*[MeSH]” AND (“regional* [all fields]” OR “rural* [all fields]” OR “remote*” [all fields])  “social mission*[Title/Abstract” AND (“regional* [all fields]” OR “rural* [all fields]” OR “remote*” [all fields])  “regional* [Title/Abstract]”  “rural* [Title/Abstract]”  “remote*[Title/Abstract]”  “underserved[Title/Abstract]”  “underrepresented[Title/Abstract]”  “under?represented[Title/Abstract]”  “Widening access [Title/Abstract]”  “Widening participation [Title/Abstract]”  “non-traditional [Title/Abstract]”  “community?based [Title/Abstract]”  “community?engaged [Title/Abstract]”  “Resource-limited settings [MeSH]”  “Place-based [Title/Abstract]” |  |
