# Supplementary material for: Medical school admission processes to target rural applicants: an international scoping review and mapping of Australian practices
Source: BMC Med Educ. 2025 May 6;25:659. doi: 10.1186/s12909-025-07234-3 (PMC12057111; doi:10.1186/s12909-025-07234-3)
Supplement: Supplementary file 3 — Supplementary Material 3 [file 12909_2025_7234_MOESM3_ESM.docx]

**Supplementary Table 3.** Complete search strategy for each database

| **Database** | **Search** | **Number of results** |
| --- | --- | --- |
| PubMed | (((((((medical schools[MeSH Terms]) OR (medical students[MeSH Terms])) OR (undergraduate medical education[MeSH Terms])) OR (undergraduate medical training[Title/Abstract])) OR (doctor of medicine[Title/Abstract])) OR (bachelor of medicine[Title/Abstract])) OR (bachelor of surgery[Title/Abstract])) OR (doctor of medicine[Title/Abstract] AND surgery[Title/Abstract]) | 82,373 |
|  | ((((((((((((((((((((((((((((((((((((((((((((((((applicant*[Title/Abstract]) OR (application*[Title/Abstract])) OR (recruit*[Title/Abstract])) OR (select*[Title/Abstract])) OR (admit*[Title/Abstract])) OR (admission*[Title/Abstract])) OR (university clinical aptitude test[Title/Abstract])) OR (ucat[Title/Abstract])) OR (undergraduate medicine[Title/Abstract] AND health sciences admission test[Title/Abstract])) OR (umat[Title/Abstract])) OR (multiple mini-interview*[Title/Abstract])) OR (MMI[Title/Abstract])) OR (interview*[Title/Abstract])) OR (personal qualit*[Title/Abstract])) OR (grade point average*[Title/Abstract])) OR (gpa[Title/Abstract])) OR (situational judgement[Title/Abstract])) OR (casper[Title/Abstract])) OR (quota*[Title/Abstract])) OR (graduate medical school admissions test[Title/Abstract])) OR (gamsat[Title/Abstract])) OR (australian tertiary admission rank[Title/Abstract])) OR (atar[Title/Abstract])) OR (overall position[Title/Abstract])) OR (op[Title/Abstract])) OR (universities admission index[Title/Abstract])) OR (uai[Title/Abstract])) OR (tertiary entrance[Title/Abstract])) OR (ter[Title/Abstract])) OR (psychometric test*[Title/Abstract])) OR (written statement*[Title/Abstract])) OR (written application*[Title/Abstract])) OR (personal statement*[Title/Abstract])) OR (subquota*[Title/Abstract])) OR (sub quota*[Title/Abstract])) OR (bonus*[Title/Abstract])) OR (adjust*[Title/Abstract])) OR (score*[Title/Abstract])) OR (standardised test*[Title/Abstract])) OR (weighted average mark[Title/Abstract])) OR (weighted?grade point average[Title/Abstract])) OR (weighted?gpa[Title/Abstract])) OR (medical admissions questionnaire[Title/Abstract])) OR (MAQ[Title/Abstract])) OR (Rank*[Title/Abstract])) OR (admissions test*[Title/Abstract])) OR (weighted mark[Title/Abstract])) OR (multiple skills assessment[Title/Abstract])) OR (msa[Title/Abstract]) | 6,977,467 |
|  | (((((((((((((regional*[Title/Abstract]) OR (rural*[Title/Abstract])) OR (remote*[Title/Abstract])) OR (underserved[Title/Abstract])) OR (underrepresented[Title/Abstract])) OR (under?represented[Title/Abstract])) OR (widening access[Title/Abstract])) OR (widening participation[Title/Abstract])) OR (non-traditional[Title/Abstract])) OR (community?based[Title/Abstract])) OR (community?engaged[Title/Abstract])) OR (resource-limited settings[MeSH Terms])) OR (place-based[Title/Abstract])) OR (((((social* accountab*[Title/Abstract]) OR (social* responsib*[Title/Abstract])) OR (social responsibilit*[MeSH Terms])) OR (social mission*[Title/Abstract])) AND (((regional*) OR (rural*)) OR (remote*))) | 722,489 |
|  | ((((((((((medical schools[MeSH Terms]) OR (medical students[MeSH Terms])) OR (undergraduate medical education[MeSH Terms])) OR (undergraduate medical training[Title/Abstract])) OR (doctor of medicine[Title/Abstract])) OR (bachelor of medicine[Title/Abstract])) OR (bachelor of surgery[Title/Abstract])) OR (doctor of medicine[Title/Abstract] AND surgery[Title/Abstract])) AND (((((((((((((((((((((((((((((((((((((((((((((((((applicant*[Title/Abstract]) OR (application*[Title/Abstract])) OR (recruit*[Title/Abstract])) OR (select*[Title/Abstract])) OR (admit*[Title/Abstract])) OR (admission*[Title/Abstract])) OR (university clinical aptitude test[Title/Abstract])) OR (ucat[Title/Abstract])) OR (undergraduate medicine[Title/Abstract] AND health sciences admission test[Title/Abstract])) OR (umat[Title/Abstract])) OR (multiple mini-interview*[Title/Abstract])) OR (MMI[Title/Abstract])) OR (interview*[Title/Abstract])) OR (personal qualit*[Title/Abstract])) OR (grade point average*[Title/Abstract])) OR (gpa[Title/Abstract])) OR (situational judgement[Title/Abstract])) OR (casper[Title/Abstract])) OR (quota*[Title/Abstract])) OR (graduate medical school admissions test[Title/Abstract])) OR (gamsat[Title/Abstract])) OR (australian tertiary admission rank[Title/Abstract])) OR (atar[Title/Abstract])) OR (overall position[Title/Abstract])) OR (op[Title/Abstract])) OR (universities admission index[Title/Abstract])) OR (uai[Title/Abstract])) OR (tertiary entrance[Title/Abstract])) OR (ter[Title/Abstract])) OR (psychometric test*[Title/Abstract])) OR (written statement*[Title/Abstract])) OR (written application*[Title/Abstract])) OR (personal statement*[Title/Abstract])) OR (subquota*[Title/Abstract])) OR (sub quota*[Title/Abstract])) OR (bonus*[Title/Abstract])) OR (adjust*[Title/Abstract])) OR (score*[Title/Abstract])) OR (standardised test*[Title/Abstract])) OR (weighted average mark[Title/Abstract])) OR (weighted?grade point average[Title/Abstract])) OR (weighted?gpa[Title/Abstract])) OR (medical admissions questionnaire[Title/Abstract])) OR (MAQ[Title/Abstract])) OR (Rank*[Title/Abstract])) OR (admissions test*[Title/Abstract])) OR (weighted mark[Title/Abstract])) OR (multiple skills assessment[Title/Abstract])) OR (msa[Title/Abstract]))) AND ((((((((((((((regional*[Title/Abstract]) OR (rural*[Title/Abstract])) OR (remote*[Title/Abstract])) OR (underserved[Title/Abstract])) OR (underrepresented[Title/Abstract])) OR (under?represented[Title/Abstract])) OR (widening access[Title/Abstract])) OR (widening participation[Title/Abstract])) OR (non-traditional[Title/Abstract])) OR (community?based[Title/Abstract])) OR (community?engaged[Title/Abstract])) OR (resource-limited settings[MeSH Terms])) OR (place-based[Title/Abstract])) OR (((((social* accountab*[Title/Abstract]) OR (social* responsib*[Title/Abstract])) OR (social responsibilit*[MeSH Terms])) OR (social mission*[Title/Abstract])) AND (((regional*) OR (rural*)) OR (remote*))))) AND ((((journal article[Publication Type]) OR (thesis[Title/Abstract])) OR (dissertation[Title/Abstract]))) Filters: English, Humans, from 2003 - 2024 | **1,683** |
|  | | |
| EMBASE (Elsevier) | #1 'medical school'/exp OR 'medical school' OR 'medical student'/exp OR 'medical student' OR 'undergraduate medical education'/exp OR 'undergraduate medical education' OR 'undergraduate medical training':ti,ab OR 'doctor of medicine':ti,ab OR 'bachelor of medicine':ti,ab OR 'bachelor of surgery':ti,ab OR 'doctor of medicine and surgery':ti,ab | 1,433,288 |
|  | #2 'applicant'/exp OR 'applicant' OR 'application'/exp OR 'application' OR 'recruit'/exp OR 'recruit' OR 'select*':ti,ab OR 'admit*':ti,ab OR 'admission*':ti,ab OR 'university clinical aptitude test':ti,ab OR 'ucat':ti,ab OR 'undergraduate medicine and health sciences admission test':ti,ab OR 'umat':ti,ab OR 'multiple mini interview'/exp OR 'multiple mini interview' OR 'mmi':ti,ab OR 'interview'/exp OR 'interview' OR 'personal qualit*':ti,ab OR 'grade point average'/exp OR 'grade point average' OR 'gpa':ti,ab OR 'situational judgement':ti,ab OR 'casper':ti,ab OR 'quota*':ti,ab OR 'graduate medical school admissions test':ti,ab OR 'gamsat':ti,ab OR 'australian tertiary admission rank':ti,ab OR 'atar':ti,ab OR 'overall position':ti,ab OR 'op':ti,ab OR 'universities admission index':ti,ab OR 'uai':ti,ab OR 'tertiary entrance':ti,ab OR 'ter':ti,ab OR 'psychometry'/exp OR 'psychometry' OR 'written statement*':ti,ab OR 'written application*':ti,ab OR 'personal statement*':ti,ab OR 'subquota*':ti,ab OR 'sub quota*':ti,ab OR 'bonus*':ti,ab OR 'adjust*':ti,ab OR 'score'/exp OR 'score' OR 'standardised test*':ti,ab OR 'weighted average mark':ti,ab OR 'weighted?grade point average':ti,ab OR 'weighted?gpa':ti,ab OR 'medical admissions questionnaire':ti,ab OR 'maq':ti,ab OR 'rank*':ti,ab OR 'admissions test*':ti,ab OR 'weighted mark':ti,ab OR 'multiple skills assessment':ti,ab OR 'msa':ti,ab | 8,012,438 |
|  | #3 'social responsibility'/exp OR 'social responsibility' OR 'social mission*':ti,ab  #4 'regional*' OR 'rural*' OR 'remote*'  #5 #3 AND #4  #6 'regional*':ti,ab OR 'rural*':ti,ab OR 'remote*':ti,ab OR 'underserved':ti,ab OR 'underrepresented':ti,ab OR 'under?represented':ti,ab OR 'widening access':ti,ab OR 'widening participation':ti,ab OR 'non-traditional':ti,ab OR 'community?based':ti,ab OR 'community?engaged':ti,ab OR 'resource limited setting'/exp OR 'resource limited setting' OR 'place-based':ti,ab  #7 #5 OR #6 | 923,517 |
|  | #8 #1 AND #2 AND #7 AND [embase]/lim NOT ([embase]/lim AND [medline]/lim) AND 'article'/it AND [english]/lim AND [humans]/lim AND (2003:py OR 2004:py OR 2005:py OR 2006:py OR 2007:py OR 2008:py OR 2009:py OR 2010:py OR 2011:py OR 2012:py OR 2013:py OR 2014:py OR 2015:py OR 2016:py OR 2017:py OR 2018:py OR 2019:py OR 2020:py OR 2021:py OR 2022:py OR 2023:py OR 2024:py) | **1,029** |
|  | | |
| Scopus | 1 TITLE-ABS ( "medical school*" ) OR TITLE-ABS ( "medical student*" ) OR TITLE-ABS ( "undergraduate medical education" ) OR TITLE-ABS ( "undergraduate medical training" ) OR TITLE-ABS ( "doctor of medicine" ) OR TITLE-ABS ( "bachelor of medicine" ) OR TITLE-ABS ( "bachelor of surgery" ) OR TITLE-ABS ( "doctor of medicine and surgery" ) | 108,147 |
|  | 2 TITLE-ABS ( applicant* ) OR TITLE-ABS ( application* ) OR TITLE-ABS ( recruit* ) OR TITLE-ABS ( select* ) OR TITLE-ABS ( admit* ) OR TITLE-ABS ( admission* ) OR TITLE-ABS ( "university clinical aptitude test" ) OR TITLE-ABS ( ucat ) OR TITLE-ABS ( "undergraduate medicine and health sciences admission test" ) OR TITLE-ABS ( umat ) OR TITLE-ABS ( "multiple mini-interview*" ) OR TITLE-ABS ( mmi ) OR TITLE-ABS ( interview* ) OR TITLE-ABS ( "personal qualit*" ) OR TITLE-ABS ( "grade point average*" ) OR TITLE-ABS ( gpa ) OR TITLE-ABS ( "situational judgement" ) OR TITLE-ABS ( casper ) OR TITLE-ABS ( quota* ) OR TITLE-ABS ( "graduate medical school admissions test" ) OR TITLE-ABS ( gamsat ) OR TITLE-ABS ( "australian tertiary admission rank" ) OR TITLE-ABS ( atar ) OR TITLE-ABS ( "overall position" ) OR TITLE-ABS ( op ) OR TITLE-ABS ( "universities admission index" ) OR TITLE-ABS ( uai ) OR TITLE-ABS ( "tertiary entrance" ) OR TITLE-ABS ( ter ) OR TITLE-ABS ( "psychometric test*" ) OR TITLE-ABS ( "written statement*" ) OR TITLE-ABS ( "written application*" ) OR TITLE-ABS ( "personal statement*" ) OR TITLE-ABS ( subquota* ) OR TITLE-ABS ( "sub?quota*" ) OR TITLE-ABS ( bonus* ) OR TITLE-ABS ( adjust* ) OR TITLE-ABS ( score* ) OR TITLE-ABS ( "standardised test*" ) OR TITLE-ABS ( "weighted average mark" ) OR TITLE-ABS ( "weighted?grade point average" ) OR TITLE-ABS ( "weighted?GPA" ) OR TITLE-ABS ( "medical admissions questionnaire" ) OR TITLE-ABS ( maq ) OR TITLE-ABS ( rank* ) OR TITLE-ABS ( "admissions test*" ) OR TITLE-ABS ( "weighted mark" ) OR TITLE-ABS ( "multiple skills assessment" ) OR TITLE-ABS ( msa ) | 18,229,553 |
|  | 3 TITLE-ABS ( "social* accountab*" ) OR TITLE-ABS ( "social* responsib*" ) OR TITLE-ABS ( "social mission*" )  4 ALL ( regional* ) OR ALL ( rural* ) OR ALL ( remote* )  5 ( TITLE-ABS ( "social* accountab*" ) OR TITLE-ABS ( "social* responsib*" ) OR TITLE-ABS ( "social mission*" ) ) AND ( ALL ( regional* ) OR ALL ( rural* ) OR ALL ( remote* ) )  6 TITLE-ABS ( regional* ) OR TITLE-ABS ( rural* ) OR TITLE-ABS ( remote* ) OR TITLE-ABS ( underserved ) OR TITLE-ABS ( underrepresented ) OR TITLE-ABS ( under?represented ) OR TITLE-ABS ( "widening access" ) OR TITLE-ABS ( "widening participation" ) OR TITLE-ABS ( non-traditional ) OR TITLE-ABS ( community?based ) OR TITLE-ABS ( community?engaged ) OR TITLE-ABS ( "resource-limited setting*" ) OR TITLE-ABS ( place-based )  7 ( ( TITLE-ABS ( "social* accountab*" ) OR TITLE-ABS ( "social* responsib*" ) OR TITLE-ABS ( "social mission*" ) ) AND ( ALL ( regional* ) OR ALL ( rural* ) OR ALL ( remote* ) ) ) OR ( TITLE-ABS ( regional* ) OR TITLE-ABS ( rural* ) OR TITLE-ABS ( remote* ) OR TITLE-ABS ( underserved ) OR TITLE-ABS ( underrepresented ) OR TITLE-ABS ( under?represented ) OR TITLE-ABS ( "widening access" ) OR TITLE-ABS ( "widening participation" ) OR TITLE-ABS ( non-traditional ) OR TITLE-ABS ( community?based ) OR TITLE-ABS ( community?engaged ) OR TITLE-ABS ( "resource-limited setting*" ) OR TITLE-ABS ( place-based ) ) | 2,098,810 |
|  | 8 SUBJAREA ( medi ) | 29,223,930 |
|  | 9 ( TITLE-ABS ( "medical school*" ) OR TITLE-ABS ( "medical student*" ) OR TITLE-ABS ( "undergraduate medical education" ) OR TITLE-ABS ( "undergraduate medical training" ) OR TITLE-ABS ( "doctor of medicine" ) OR TITLE-ABS ( "bachelor of medicine" ) OR TITLE-ABS ( "bachelor of surgery" ) OR TITLE-ABS ( "doctor of medicine and surgery" ) ) AND ( TITLE-ABS ( applicant* ) OR TITLE-ABS ( application* ) OR TITLE-ABS ( recruit* ) OR TITLE-ABS ( select* ) OR TITLE-ABS ( admit* ) OR TITLE-ABS ( admission* ) OR TITLE-ABS ( "university clinical aptitude test" ) OR TITLE-ABS ( ucat ) OR TITLE-ABS ( "undergraduate medicine and health sciences admission test" ) OR TITLE-ABS ( umat ) OR TITLE-ABS ( "multiple mini-interview*" ) OR TITLE-ABS ( mmi ) OR TITLE-ABS ( interview* ) OR TITLE-ABS ( "personal qualit*" ) OR TITLE-ABS ( "grade point average*" ) OR TITLE-ABS ( gpa ) OR TITLE-ABS ( "situational judgement" ) OR TITLE-ABS ( casper ) OR TITLE-ABS ( quota* ) OR TITLE-ABS ( "graduate medical school admissions test" ) OR TITLE-ABS ( gamsat ) OR TITLE-ABS ( "australian tertiary admission rank" ) OR TITLE-ABS ( atar ) OR TITLE-ABS ( "overall position" ) OR TITLE-ABS ( op ) OR TITLE-ABS ( "universities admission index" ) OR TITLE-ABS ( uai ) OR TITLE-ABS ( "tertiary entrance" ) OR TITLE-ABS ( ter ) OR TITLE-ABS ( "psychometric test*" ) OR TITLE-ABS ( "written statement*" ) OR TITLE-ABS ( "written application*" ) OR TITLE-ABS ( "personal statement*" ) OR TITLE-ABS ( subquota* ) OR TITLE-ABS ( "sub?quota*" ) OR TITLE-ABS ( bonus* ) OR TITLE-ABS ( adjust* ) OR TITLE-ABS ( score* ) OR TITLE-ABS ( "standardised test*" ) OR TITLE-ABS ( "weighted average mark" ) OR TITLE-ABS ( "weighted?grade point average" ) OR TITLE-ABS ( "weighted?GPA" ) OR TITLE-ABS ( "medical admissions questionnaire" ) OR TITLE-ABS ( maq ) OR TITLE-ABS ( rank* ) OR TITLE-ABS ( "admissions test*" ) OR TITLE-ABS ( "weighted mark" ) OR TITLE-ABS ( "multiple skills assessment" ) OR TITLE-ABS ( msa ) ) AND ( ( ( TITLE-ABS ( "social* accountab*" ) OR TITLE-ABS ( "social* responsib*" ) OR TITLE-ABS ( "social mission*" ) ) AND ( ALL ( regional* ) OR ALL ( rural* ) OR ALL ( remote* ) ) ) OR ( TITLE-ABS ( regional* ) OR TITLE-ABS ( rural* ) OR TITLE-ABS ( remote* ) OR TITLE-ABS ( underserved ) OR TITLE-ABS ( underrepresented ) OR TITLE-ABS ( under?represented ) OR TITLE-ABS ( "widening access" ) OR TITLE-ABS ( "widening participation" ) OR TITLE-ABS ( non-traditional ) OR TITLE-ABS ( community?based ) OR TITLE-ABS ( community?engaged ) OR TITLE-ABS ( "resource-limited setting*" ) OR TITLE-ABS ( place-based ) ) ) AND ( SUBJAREA ( medi ) ) | 1,904 |
|  | ( TITLE-ABS ( "medical school*" ) OR TITLE-ABS ( "medical student*" ) OR TITLE-ABS ( "undergraduate medical education" ) OR TITLE-ABS ( "undergraduate medical training" ) OR TITLE-ABS ( "doctor of medicine" ) OR TITLE-ABS ( "bachelor of medicine" ) OR TITLE-ABS ( "bachelor of surgery" ) OR TITLE-ABS ( "doctor of medicine and surgery" ) ) AND ( TITLE-ABS ( applicant* ) OR TITLE-ABS ( application* ) OR TITLE-ABS ( recruit* ) OR TITLE-ABS ( select* ) OR TITLE-ABS ( admit* ) OR TITLE-ABS ( admission* ) OR TITLE-ABS ( "university clinical aptitude test" ) OR TITLE-ABS ( ucat ) OR TITLE-ABS ( "undergraduate medicine and health sciences admission test" ) OR TITLE-ABS ( umat ) OR TITLE-ABS ( "multiple mini-interview*" ) OR TITLE-ABS ( mmi ) OR TITLE-ABS ( interview* ) OR TITLE-ABS ( "personal qualit*" ) OR TITLE-ABS ( "grade point average*" ) OR TITLE-ABS ( gpa ) OR TITLE-ABS ( "situational judgement" ) OR TITLE-ABS ( casper ) OR TITLE-ABS ( quota* ) OR TITLE-ABS ( "graduate medical school admissions test" ) OR TITLE-ABS ( gamsat ) OR TITLE-ABS ( "australian tertiary admission rank" ) OR TITLE-ABS ( atar ) OR TITLE-ABS ( "overall position" ) OR TITLE-ABS ( op ) OR TITLE-ABS ( "universities admission index" ) OR TITLE-ABS ( uai ) OR TITLE-ABS ( "tertiary entrance" ) OR TITLE-ABS ( ter ) OR TITLE-ABS ( "psychometric test*" ) OR TITLE-ABS ( "written statement*" ) OR TITLE-ABS ( "written application*" ) OR TITLE-ABS ( "personal statement*" ) OR TITLE-ABS ( subquota* ) OR TITLE-ABS ( "sub?quota*" ) OR TITLE-ABS ( bonus* ) OR TITLE-ABS ( adjust* ) OR TITLE-ABS ( score* ) OR TITLE-ABS ( "standardised test*" ) OR TITLE-ABS ( "weighted average mark" ) OR TITLE-ABS ( "weighted?grade point average" ) OR TITLE-ABS ( "weighted?GPA" ) OR TITLE-ABS ( "medical admissions questionnaire" ) OR TITLE-ABS ( maq ) OR TITLE-ABS ( rank* ) OR TITLE-ABS ( "admissions test*" ) OR TITLE-ABS ( "weighted mark" ) OR TITLE-ABS ( "multiple skills assessment" ) OR TITLE-ABS ( msa ) ) AND ( ( ( TITLE-ABS ( "social* accountab*" ) OR TITLE-ABS ( "social* responsib*" ) OR TITLE-ABS ( "social mission*" ) ) AND ( ALL ( regional* ) OR ALL ( rural* ) OR ALL ( remote* ) ) ) OR ( TITLE-ABS ( regional* ) OR TITLE-ABS ( rural* ) OR TITLE-ABS ( remote* ) OR TITLE-ABS ( underserved ) OR TITLE-ABS ( underrepresented ) OR TITLE-ABS ( under?represented ) OR TITLE-ABS ( "widening access" ) OR TITLE-ABS ( "widening participation" ) OR TITLE-ABS ( non-traditional ) OR TITLE-ABS ( community?based ) OR TITLE-ABS ( community?engaged ) OR TITLE-ABS ( "resource-limited setting*" ) OR TITLE-ABS ( place-based ) ) ) AND ( SUBJAREA ( medi ) ) AND PUBYEAR > 2002 AND PUBYEAR < 2025 AND ( LIMIT-TO ( DOCTYPE , "ar" ) ) AND ( LIMIT-TO ( EXACTKEYWORD , "Human" ) ) AND ( LIMIT-TO ( LANGUAGE , "English" ) ) | **1,136** |
|  | | |
| Web of Science | #1 (((((((((((((((TI=(medical school*)) OR AB=(medical school*)) OR TI=(medical student*)) OR AB=(medical student*)) OR TI=(undergraduate medical education)) OR AB=(undergraduate medical education)) OR TI=(undergraduate medical training)) OR AB=(undergraduate medical training)) OR TI=(doctor of medicine)) OR AB=(doctor of medicine)) OR TI=(bachelor of medicine)) OR AB=(bachelor of medicine)) OR TI=(bachelor of surgery)) OR AB=(bachelor of surgery)) OR TI=(doctor of medicine and surgery)) OR AB=(doctor of medicine and surgery) | 145,253 |
|  | #2 ((((((((((((((((((((((((((((((((((((((((((((((((TI=(applicant*)) OR TI=(application*)) OR TI=(recruit*)) OR TI=(select*)) OR TI=(admit*)) OR TI=(admission*)) OR TI=(university clinical aptitude test)) OR TI=(ucat)) OR TI=(undergraduate medicine and health sciences admission test)) OR TI=(umat)) OR TI=(multiple mini-interview*)) OR TI=(mmi)) OR TI=(interview*)) OR TI=(personal qualit*)) OR TI=(grade point average*)) OR TI=(gpa)) OR TI=(situational judgement)) OR TI=(casper)) OR TI=(quota*)) OR TI=(graduate medical school admissions test)) OR TI=(gamsat)) OR TI=(australian tertiary admission rank)) OR TI=(atar)) OR TI=(overall position)) OR TI=(op)) OR TI=(universities admission index)) OR TI=(uai)) OR TI=(tertiary entrance)) OR TI=(ter)) OR TI=(psychometric test*)) OR TI=(written statement*)) OR TI=(written application*)) OR TI=(personal statement*)) OR TI=(subquota*)) OR TI=(sub quota*)) OR TI=(bonus*)) OR TI=(adjust*)) OR TI=(score*)) OR TI=(standardised test*)) OR TI=(weighted average mark)) OR TI=(weighted?grade point average)) OR TI=(weighted?gpa)) OR TI=(medical admissions questionnaire)) OR TI=(maq)) OR TI=(rank*)) OR TI=(admissions test*)) OR TI=(weighted mark)) OR TI=(multiple skills assessment)) OR TI=(msa) | 2,808,291 |
|  | #3 ((TI=(social*accountab*)) OR TI=(social* responsib*)) OR TI=(social mission*)  #4 ((ALL=(regional*)) OR ALL=(rural*)) OR ALL=(remote*)  #5 #4 AND #3  #6 ((((((((((((TI=(regional*)) OR TI=(rural*)) OR TI=(remote*)) OR TI=(underserved)) OR TI=(underrepresented)) OR TI=(under?represented)) OR TI=(widening access)) OR TI=(widening participation)) OR TI=(non-traditional)) OR TI=(community?based)) OR TI=(community?engaged)) OR TI=(resource-limited setting*)) OR TI=(place-based)  #7 #6 OR #5 | 515,523 |
|  | #8 #1 AND #2 AND #7 | 189 |
|  | #9 #8 AND 2022 or 2023 or 2021 or 2020 or 2019 or 2017 or 2018 or 2016 or 2015 or 2013 or 2012 or 2011 or 2014 or 2010 or 2009 or 2008 or 2007 or 2006 or 2005 or 2004 or 2003 (Publication Years) and Article (Document Types) and English (Languages) | **134** |
