# Supplementary material for: Medical school admission processes to target rural applicants: an international scoping review and mapping of Australian practices
Source: BMC Med Educ. 2025 May 6;25:659. doi: 10.1186/s12909-025-07234-3 (PMC12057111; doi:10.1186/s12909-025-07234-3)
Supplement: Supplementary file 4 — Supplementary Material 4 [file 12909_2025_7234_MOESM4_ESM.docx]

**Supplementary Table 4.** Admission requirements and processes for each pathway and medical school

| **Medical school** | **Pathway** | **Components used for interview selection and weighting** | **Components used for admission and weighting** | **Minimum requirements** | **Rural background criteria** | **Other rural criteria and quotas** | **Adjustments for rural applicants** | **How interview and admission offers are made** | **Allocation of bonded places** |
| --- | --- | --- | --- | --- | --- | --- | --- | --- | --- |
| The University of Adelaide | Domestic – Recent secondary education | 100% UCAT (sections 1-4) | 40% interview  40% ATAR  20% UCAT | 90 ATAR | 5 consecutive/10 cumulative years  RA2-5 | 50% local (state) residents interviewed | ≤5 ATAR adjustments (school-based) | Highest ranked | Separate course codes |
|  | Domestic – Higher Education Study | 100% UCAT (sections 1-4) | 40% interview  40% GPA  20% UCAT | 5.0 GPA | 5 consecutive/10 cumulative years  RA2-5 | 10 Higher education places | ≤5 ATAR adjustments (school-based) | Highest ranked | Separate course codes |
|  | International | 100% UCAT (sections 1-4) | Interview  UCAT | 90 ATAR | NA | NA | NA | Two ranking levels | NA |
|  | Aboriginal Access Scheme | All eligible applicants interviewed | Literacy/numeracy  Interview  Student success plan  Self-assessment | Resume/transcripts  Student success plan  Self-assessment | NA | NA | NA | Not reported (offers) | Not reported |
| Australian National University | Health Sciences | Meeting minimum requirements | 40% WAM  40% interview  20% written application | 5.0 GPA  ≥60% interview | 5 consecutive/10 cumulative years  MM2-7 | NA | Quota | Ranking (admission) | Not reported |
|  | Philosophy (Honours) | Not reported | 50% GPA  50% interview | ≥60% interview  Satisfactory academic progress | 5 consecutive/10 cumulative years  MM2-7 | NA | Not reported | Ranking (admission) | Not reported |
|  | Domestic | 50% GAMSAT  50% GPA | 50% interview  25% GPA  25% GAMSAT | 5.6 GPA  50 each GAMSAT section/55 overall | 5 consecutive/10 cumulative years  MM2-7 | 20 places for current students | Quota | Ranking | Not reported |
|  | International | 50% GAMSAT/MCAT  50% GPA | 50% interview  25% GPA  25% GAMSAT/MCAT | 5.0 GPA  50 each GAMSAT section/55 overall | NA | NA | NA | Ranking | NA |
|  | Aboriginal and Torres Strait Islander Pathway | 50% GAMSAT  50% GPA | 50% interview  25% GPA  25% GAMSAT | 5.0 or 5.6 GPA (conflicting information reported)  50 each GAMSAT section/50 or 55 overall (conflicting information) | NA | NA | Not reported | Ranking | Not reported |
| Bond University | Undergraduate | UCAT/GPA – ranked 1^st^  Psychometric testing – ranked 2^nd^ | MMI – ranked 3^rd^ | 96 ATAR | NA | NA | NA | Three ranking levels | NA (fee paying) |
|  | Graduate | GPA – ranked 1^st^ Psychometric testing – ranked 2^nd^ | MMI – ranked 3^rd^ | 6.0 GPA | NA | NA | NA | Three ranking levels | NA (fee paying) |
|  | Lateral | GPA – ranked 1^st^  Psychometric testing – ranked 2^nd^ | MMI – ranked 3^rd^ | 3.0 GPA (out of 4) | NA | NA | NA | Three ranking levels | NA (fee paying) |
| Charles Sturt University (CSU) and Western Sydney University (WSU) | CSU - Domestic | 100% UCAT | 75% interview  25% UCAT | 95.5 ATAR/5.4-6.5 GPA depending on degree and complete/incomplete | NA | 80% interviews local (state) applicants  Six interviews current students | NA | Highest ranked | Non-bonded places offered first (highest ranked) |
|  | CSU – Rural | 100% UCAT | 75% Interview  25% UCAT | 91.5 ATAR/4.6-6.5 GPA depending on the degree and complete/incomplete | 5 consecutive/10 cumulative years since age 5  RA2-5 | 80% interviews local (state) applicants  Six interviews current students | Separate pathway | Highest ranked | Non-bonded places offered first (highest ranked) |
|  | CSU – First Nations | Not reported | Interview  Academic records  Resume  Community involvement | HSC completed | NA | NA | NA | Not reported | Not reported |
|  | WSU - Domestic | 100% UCAT (sections may be weighted) | 75% Interview  25% UCAT | ATAR 93.5 (local residents)/95.5 (non-local residents)  GPA 4.9 – 6.0 (local)/5.4-6.5 (non-local) depending on the degree and complete/incomplete | NA | 80% interviews local (state) applicants; proportion of interviews for applicants in the local area | NA | Highest ranked | Based on final ranking |
|  | WSU – Rural | 100% UCAT (sections may be weighted) | 75% Interview  25% UCAT | 91.5 ATAR  4.9-6.0 GPA (local residents)/5.4-6.5 (non-local residents) depending on the degree and complete/incomplete | 5 consecutive/10 cumulative years since age 5  RA2-5 | Proportion of interviews for local applicants | Separate pathway | Highest ranked | Non-bonded places offered first (highest ranked) |
|  | WSU – Aboriginal and Torres Strait Islander | Not reported | Interview  Academic records  Community involvement  Resume | HSC or tertiary equivalent completed | NA | NA | NA | Not reported | Not reported |
|  | WSU - International | Meeting minimum eligibility criteria | Not reported | 93.5 ATAR  4.9-6.1 GPA depending on degree and complete/incomplete  160 on ISAT/50% in each section | NA | NA | NA | Not reported | NA |
| Curtin University | School leaver | 35% ATAR  35% Casper  30% UCAT | 40% ATAR  40% interview  20% UCAT | 95 ATAR | 5 consecutive/10 cumulative years since year 1  MM2-7 and same state as the program | Equity places available | ≤5 ATAR adjustments | Highest ranked | Not reported |
|  | Non-school leaver course switcher | 35% ATAR  35% Casper  30% UCAT | 40% ATAR  40% Interview  20% UCAT | 80% course weighted average | 5 consecutive/10 cumulative years since year 1  MM2-7 and same state as the program | Equity places available | ≤5 ATAR adjustments | Highest ranked | Not reported |
|  | Graduates | 35% ATAR  35% Casper  30% UCAT | 40% ATAR  40% Interview  20% UCAT | 92 ATAR | 5 consecutive/10 cumulative years since year 1  MM2-7 and same state as the program | Equity places available | ≤5 ATAR adjustments | Highest ranked | Not reported |
|  | Aboriginal and Torres Strait Islander | NA (pre-admissions program rather than interview) | Academic performance  Written application  Pre-admissions assessment | Complete pre-medicine enabling course or at least 1 year of tertiary study | NA | NA | NA | Pre-admissions program | Not reported |
|  | International | 35% academic  35% Casper  30% UCAT | 40% academic performance  40% interview  20% UCAT | 95 ATAR (school leavers) or 92 (non-school leavers) | NA | NA | NA | Highest ranked | NA |
| Deakin University | General domestic | 50% GPA  50% GAMSAT | 50% MMI  50% GPA + GAMSAT + adjustments | 5.0 GPA  50 each GAMSAT section and overall | 5 consecutive/10 cumulative years  MM2-7 or local | NA | 4% adjustment to GPA/GAMSAT score (local/MM2)/8% for MM3-7 | Ranking | Not reported |
|  | Rural | 50% GPA + adjustments (local)  50% GPA + GAMSAT + adjustments (rural)  50% written application (all) | 50% MMI (all)  25% written application (all)  25% GPA + adjustments (local)  25% GPA + GAMSAT + adjustments (rural) | 5.0 GPA (rural)  50 each GAMSAT section and overall (rural)  NA for local applicants | 5 consecutive/10 cumulative years  MM2-7 or local | NA | 4% adjustment to GPA/GAMSAT score (local/MM2)/8% for MM3-7 | Tiers then ranking | Not reported |
|  | International | 50% GPA  50% GAMSAT/MCAT | 50% interview  25% GPA  25% GAMSAT/MCAT | 5.0 GPA  50 each GAMSAT section and overall/125 each MCAT section  50% interview | NA | NA | NA | Ranking | NA |
|  | Indigenous entry | Not reported | GPA and interview | 5.0 GPA  50% on the interview | 5 consecutive/10 cumulative years  MM2-7 or local | NA | 4% adjustment to GPA/GAMSAT score (local/MM2)/8% for MM3-7 | Ranking | Not reported |
| Flinders University | Undergraduate SA | Meeting minimum requirements | 90% ATAR  10% UCAT  Or based on interview performance (conflicting information) | 95 ATAR | 5 consecutive/10 cumulative years  RA2-5 | NA | Not reported | Unclear | Not reported |
|  | Provisional NT | NA | 90% ATAR  10% UCAT | 90 ATAR | Permanent NT address for two of the last six years or 5 years total since commencing high school | 5 places NT residents  5 places first nations NT residents  2 places for first nations | NA | Ranking within each subquota | Not reported |
|  | Graduate SA | 100% GAMSAT | 33.3% GAMSAT  33.3% GPA  33.3% interview | 5.0 GPA  50 on each GAMSAT section  Overall GAMSAT: 57 (rural sub quota), 62 (humanitarian visa holders; 64 (flinders graduates/rural), 67 flinders graduates, 76 (others)  50% interview | 5 consecutive/10 cumulative years  RA2-5 | 5 Aboriginal/Torres Strait Islander  4 humanitarian visa holders  35 rural  75% Flinders graduates | Subquotas | Ranking within each subquota | Highest ranked offered non-bonded |
|  | Graduate NT | 100% GAMSAT | 33.3% GAMSAT  33.3% GPA  33.3% interview | 5.0 GPA  50 on GAMSAT section  50% interview | Permanent NT address for two of the last six years or 5 years total since commencing high school | Priority given to NT and indigenous applicants | Not reported | Not reported (interview)  Ranked based on government priorities (indigenous NT, non-indigenous NT, non-NT indigenous, non-NT non-indigenous; offers) | All bonded |
|  | Aboriginal and Torres Strait Islander | Indigenous application | After interviews, invited to a two-week preparation course and extended learning course | 5.0 GPA  Two-week preparation course  10-12 week extended learning in science course | Not reported | NA | Not reported | Not reported | Not reported |
| Griffith University | Domestic | 50% GPA  50% GAMSAT | 50% interview  25% GAMSAT  25% GPA | 50 on each GAMSAT section and overall | 5 consecutive/10 cumulative years  MM2-7 | NA | Not reported | Highest ranked | Based on ranking/student preference |
|  | International | 50% GPA  50% GAMSAT/MCAT | Not reported | GPA 5.0  50 each GAMSAT section/123 each MCAT section | NA | NA | NA | Highest ranked | NA |
|  | Aboriginal and Torres Strait Islander | Meeting minimum requirements | Not reported | GPA 5.0  Resume  Personal statement | NA | NA | NA | Not reported | Not reported |
| James Cook University | Domestic | Academic results + online application (school leaver)  GPA (commenced tertiary study) | Online application + academic results + rurality or indigenous background + interview | NA | Five consecutive/10 cumulative years  MM2-7 | NA | Not reported | Not reported | Not reported |
|  | International | Not reported | Not reported | NA | Not reported | NA | Not reported | Not reported | NA |
| Macquarie University | General | 50% GPA  50% GAMSAT | 50% GPA  50% MMI | 5.0 GPA/65 weighted mark  50 each GAMSAT section and overall  Satisfactory personal statement | Not reported | 20 places for Bachelor of Clinical Science students/graduates | 3% bonus to weighted GPA (maximum 5 total adjustments) | Highest ranked | NA (fee-paying) |
|  | International | 50% weighted GPA  50% GAMSAT/MCAT | 50% GPA  50% MMI | 5.0 GPA/65 weighted mark  50 each GAMSAT section and overall/500 MCAT  Satisfactory personal statement | NA | NA | NA | Highest ranked | NA (fee-paying) |
|  | Indigenous Pathway | GPA  GAMSAT (optional) | Not reported | 5.0 GPA after 3% Aboriginal and Torres Strait Islander adjustment | NA | NA | NA | Case-by-case basis | NA (fee-paying) |
| The University of Melbourne | Standard (Domestic/International) | 50% GPA  50% GAMSAT/MCAT | 50% interview  25% GPA  25% GAMSAT/MCAT | 5.0 GPA  50 each GAMSAT section/492 MCAT | Not reported/NA | NA | NA | Ranking | NA |
|  | Guaranteed entry | Meet eligibility requirements | Not reported | Entered undergraduate degree with 99.9 ATAR  Pass MMI  75 WAM | NA | NA | NA | Meet minimum requirements | Not reported |
|  | Rural | 100% GPA | 70% MMI  30% GPA | 5.0 GPA  Pass MMI | Five consecutive/10 cumulative years  MM2-7 | 15 places LaTrobe graduates | NA | Ranking | All bonded |
|  | Indigenous pathway | Meet minimum requirements | Pass the MMI to be invited for a panel interview to determine entry | 5.0 GPA  Pass MMI | NA | NA | NA | Minimum requirements (MMI/panel interview)  Not reported (admission) | Not reported |
| Monash University | School leaver | UCAT or UCAT + ATAR | 33.3% ATAR  33.3% UCAT  33.3% interview | 90 ATAR | Five consecutive/10 cumulative years  MM2-7 | NA | Not reported | Not reported | Not reported |
|  | Graduate domestic | 100% weighted mark | 60% MMI  40% academic record | 70 WAM | Five consecutive/10 cumulative years  MM2-7 | NA | Not reported | Not reported | Not reported |
|  | Graduate international | 100% GPA | Not reported | 6.0 GPA | NA | NA | NA | Not reported | NA |
| The University of Newcastle and the University of New England | General domestic | 100% UCAT (sections 1-4) | Personal qualities and MSA | 94.3 ATAR/91.4 rural | Five consecutive/10 cumulative years  RA2-5 (ineligible if year 12 was completed at a metropolitan high school) | NA | Lower ATAR | Ranked at 2 stages | Not reported |
|  | International | 100% UCAT (sections 1-4) | Personal qualities and MSA | 94.3 ATAR | NA | NA | NA | Ranked at 2 stages | NA |
|  | Equity pathway | Meeting equity criteria | Not reported | Demonstrate socioeconomic disadvantage  Personal statement  94.3 ATAR/91.4 rural | Five consecutive/10 cumulative years  RA2-5 (ineligible if year 12 was completed at a metropolitan high school) | NA | Lower ATAR | Not reported | Not reported |
|  | Aboriginal and Torres Strait Islander | Meet eligibility requirements | Personal qualities and MSA | 72.55 ATAR | NA | NA | NA | Ranking and pre-entry program requirements | Not reported |
| University of Notre Dame | General Freemantle | 30% GAMSAT  30% GPA  30% Casper  10% Bonus points | 50% GAMSAT, GPA, Casper, and bonus points  50% interview | 50 each GAMSAT section and 52 overall  5.2 GPA | Come from an MM2-7 area | Bonus points for rurality and residing in the same state | Bonus points | Highest ranked | Offers are made to CSPs then BMPs, then full-fee paying |
|  | General Sydney | 30% GAMSAT  30% GPA  30% Casper  10% Bonus points | 50% GAMSAT, GPA, Casper, and bonus points  50% interview | 50 each GAMSAT section and 52 overall  5.2 GPA | Come from an MM2-7 area | NA | Bonus points | Highest ranked | Offers are made to CSPs then BMPs, then full-fee paying |
|  | International Freemantle | Not reported | Not reported | 50 each GAMSAT section and 52 overall/500 MCAT  5.2 GPA | NA | NA | NA | Ranking | NA |
|  | International Sydney | Not reported | Not reported | 50 each GAMSAT section and 52 overall/500 MCAT  5.2 GPA | NA | NA | NA | Ranking | NA |
|  | Aboriginal entry - Freemantle | Not reported | Application  Prior academic achievement  Interview | Not reported | 5 consecutive/10 cumulative years  Rural/regional Australia | NA | NA | Not reported | Not reported |
|  | Aboriginal entry - Sydney | Not reported | Not reported | GAMSAT  GPA considered | NA | NA | NA | Not reported | Not reported |
|  | Priority pathway | Not reported | GPA  GAMSAT  Casper Test  Interview | Minimum GPA  Minimum GAMSAT  Complete Casper and interview (unclear if same as general entry) | NA | NA | NA | Not reported | Not reported |
|  | Assured pathway | 100% UCAT | Not reported | Minimum ATAR  2.5 GPA each semester | NA | NA | NA | Ranking (interview)  Not reported (offers) | Not reported |
| The University of Sydney | Undergraduate | NA | Written assessment followed by a panel discussion | 99.95 ATAR/90 Aboriginal and Torres Strait Islander | RA2-5 or recent school leaver (RA2-5 school) | NA | Lower ATAR | Not reported | Not reported |
|  | Graduate metropolitan | NA | GAMSAT (each section)  GPA | 5.0 GPA/4.5 rural  50 on each GAMSAT section | 5 consecutive/10 cumulative years  RA2-5 | NA | Lower GPA | Ranking | Separate ranked lists |
|  | Graduate rural | NA | GAMSAT (each section)  GPA | 5.0 GPA/4.5 rural  50 on each GAMSAT section | 5 consecutive/10 cumulative years  RA2-5 | NA | Lower GPA | Ranking | Separate ranked lists |
|  | Graduate Indigenous Facilitated Entry | NA | GPA  GAMSAT  Resume | 4.5 GPA preferred  50 on each GAMSAT section preferred  Passed section 3 of the GAMSAT | NA | NA | NA | Assessed on an individual basis by the committee | Not reported |
|  | Graduate international | NA | GAMSAT/MCAT | 5.0 GPA  50 on each GAMSAT section/500 MCAT | NA | NA | NA | Ranking | NA |
| The University of New South Wales | Domestic | Selection rank (ATAR + adjustments)  UCAT | ATAR  UCAT  Interview | 96 ATAR  50^th^ percentile on UCAT (sections 1-4) | NA | NA | NA | Ranking | Highest ranked offered unbonded |
|  | International | Predicted/actual academic merit | UCAT  Academic merit  Interview | 96 ATAR  50th percentile on UCAT (sections 1-4) | NA | NA | NA | Ranking | NA |
|  | Rural | Selection rank  UCAT | ATAR  UCAT  Interview  Rural rating | 91 ATAR  50th percentile on UCAT (sections 1-4) | 5 consecutive/10 cumulative years between ages 5 and 18  MM2-7 | NA | Lower ATAR | Ranking | Highest ranked offered unbonded |
|  | Gateway | Selection rank  UCAT | ATAR  UCAT  Interview | 91 ATAR  50th percentile on UCAT (sections 1-4)  Meet SES criteria | NA | NA | NA | Ranking | Highest ranked offered unbonded |
|  | Lateral | 50% WAM  50% UCAT (sections 1-4) | WAM  UCAT  Interview | 50th percentile on UCAT (sections 1-4) | NA | NA | NA | Not reported (interview)  Ranking (admission) | Not reported |
|  | Indigenous Entry Scheme | NA (selected to pre-medicine program) | Based on performance during the pre-medicine program | Not reported | NA | NA | NA | Not reported | Not reported |
| The University of Queensland | Provisional entry - Brisbane | 100% UCAT | ATAR  MMI  UCAT | 95 ATAR | 5 consecutive/10 cumulative years  MM2-7 | 50% interview invitations to local (state) applicants | 2 adjustments to selection rank | Ranking | Separate course codes |
|  | Provisional entry - Darling-Downs/SouthWest | 100% UCAT sections 1-4 | Not reported | 95 ATAR | 5 consecutive/10 cumulative years  MM2-7 | Aim for equal number of interviews for local applicants, other rural applicants, and all other applicants | 2 adjustments to selection rank or 3 if local | Tiers (interview) and ranking | Separate course codes |
|  | Provisional – Central Queensland/Wide Bay | 100% UCAT sections 1-4 | 50% MMI  25% ATAR  25% UCAT | 95 ATAR | 5 consecutive/10 cumulative years  MM2-7 | Equal number of interviews for local applicants, other rural applicants, and all other applicants | 2 adjustments to selection rank or 3 if local | Tiers (interview) and ranking | Separate course codes |
|  | Provisional - Aboriginal and Torres Strait Islander Brisbane | Determined by selection committee | Not reported | Pass prerequisites  UCAT encouraged  Resume  Personal statement | NA | NA | NA | Selection committee | Separate course codes |
|  | Provisional - Aboriginal and Torres Strait Islander - Central Queensland/Wide Bay | All applicants complete a semi-structured interview to determine whether they progress to MMI | MMI  Recommendation from the Office of Indigenous Engagement | Minimum MMI score | NA | NA | NA | Semi-structured interview and recommendation from the Office of Indigenous Engagement | Separate course codes |
|  | Provisional - International | 100% UCAT | ATAR  UCAT  Interview | 95 ATAR | NA | NA | NA | Ranking | NA |
|  | Graduate - Brisbane | 50% GAMSAT  50% GPA | 50% interview  25% GAMSAT  25% GPA | 5.0 GPA  50 each GAMSAT section | 5 consecutive/10 cumulative years  MM2-7 | NA | Two adjustments to unweighted GAMSAT | Ranking | Non-bonded offered first; rural background places filled with bonded and non-bonded places |
|  | Graduate – Darling-Downs/SouthWest | 50% GAMSAT  50% GPA | 50% interview  25% GAMSAT  25% GPA | 5.0 GPA  50 each GAMSAT section | 5 consecutive/10 cumulative years  MM2-7 | Equal number of interviews for local applicants, other rural applicants, and all other applicants | Two adjustments to unweighted GAMSAT | Tiers (interview) and ranking | Non-bonded offered first; rural background places filled with bonded and non-bonded places |
|  | Graduate – Central Queensland/Wide Bay | 50% GAMSAT  50% GPA | 50% interview  25% GAMSAT  25% GPA | 5.0 GPA  50 each GAMSAT section | 5 consecutive/10 cumulative years  MM2-7 | Equal number of interviews for local applicants, other rural applicants, and all other applicants | Two adjustments to unweighted GAMSAT | Tiers (interview) and ranking | Non-bonded offered first; rural background places filled with bonded and non-bonded places |
|  | Graduate - International | Not reported | Not reported | 5.0 GPA  50 on each GAMSAT section/504 MCAT | NA | NA | NA | Not reported | NA |
|  | Graduate - Ochsner | MCAT  GPA | MCAT  GPA  MMI | Final year or completed Bachelor’s degree in the last 10 years and B average  504 MCAT | NA | NA | NA | Ranking | NA |
|  | Graduate - Aboriginal and Torres Strait Islander | Determined by selection committee | Not reported | GAMSAT encouraged  Resume  Transcripts  Personal statement | NA | NA | NA | Selection committee | Not reported |
| The University of Tasmania | Domestic | NA | ATAR/GPA  UCAT/GAMSAT | 95 ATAR | 5 consecutive/10 cumulative years  RA2-5 | 75% local (state) applicants admitted | ≤5 ATAR adjustments | Ranking | Not reported |
|  | Rural | NA | ATAR/GPA  UCAT/GAMSAT | 95 ATAR | 5 consecutive/10 cumulative years  RA2-5 | 75% local (state) applicants admitted | ≤5 ATAR adjustments | Ranking | Not reported |
|  | International | NA | ATAR  UCAT/MCAT | 90 ATAR | NA | NA | NA | Not reported | NA |
|  | Aboriginal and Torres Strait Islander | Not reported | Personal statement  ATAR  Qualifications  Referee report  Interview | NA | NA | NA | NA | Not reported | Not reported |
| The University of Western Australia | School leaver - Domestic | 100% UCAT | Interview – 50% non-rural/27.5% rural  ATAR – 30% non-rural/22.5% rural  UCAT – 20% non-rural/15% rural  Rural rating – 25% rural | 98 ATAR | 5 consecutive/10 cumulative years  MM2-7 | Majority of places local (state) residents | ≤5 ATAR adjustments | Ranking | Based on ranking |
|  | School leaver - International | Not reported | 50% interview  30% ATAR  20% ISAT | 98 ATAR | NA | NA | NA | Ranking | NA |
|  | School leaver - Indigenous | Not reported | GPA  Interview | 90 ATAR | NA | NA | NA | Not reported (interview)  Ranking (offers) | Not reported |
|  | Graduate - Domestic | GPA  GAMSAT | Interview – 50% no-rural/37.5% rural  GPA – 30% non-rural/22.5% rural  GAMSAT – 20% non-rural, 15% rural  Rural rating – 15% rural | 5.5 GPA  55 GAMSAT and 50 in each section | 5 consecutive/10 cumulative years  MM2-7 | NA | Different weightings of admission components | Ranking | Non-bonded offered first |
|  | Graduate - International | GPA  GAMSAT/MCAT | 50% interview  30% GPA  20% GAMSAT | 5.5 GPA  50 each GAMSAT section and 55 overall/124 each MCAT section and 500 overall | NA | NA | NA | Ranking | NA |
|  | Graduate - Indigenous | Not reported | GPA  Interview | 5.0 GPA | NA | NA | NA | Not reported (interview)  Ranking (offers) | Not reported |
| The University of Wollongong | General | 50% GAMSAT  50% portfolio | 50% portfolio  25% interview  25% Casper | GPA and Casper test used as hurdles prior to interview selection  5.5 weighted GPA  50 each GAMSAT section and overall | Living/studying in an MM2-7 area | NA | NA | Ranking | According to rank and preference |
|  | Rural | 50% GAMSAT  50% portfolio | 50% portfolio  25% interview  25% Casper | GPA and Casper test used as hurdles prior to interview selection  5.5 weighted GPA  50 GAMSAT overall and in each section | Living/studying in an MM2-7 area | NA | Separate pathway | Ranking | According to rank and preference |
|  | International | GPA  Casper  GAMSAT  Portfolio | GPA  GAMSAT/MCAT  Casper Test  Portfolio  Interview | 5.5 GPA  50 each GAMSAT section and overall/123 each MCAT section and 495 overall | NA | NA | NA | Ranking | NA |
|  | Aboriginal and Torres Strait Islander | Meeting minimum requirements | Weighted GPA  MMI  Community interview | 5.5 GPA  50 GAMSAT overall and in each section (may be exempt)  Admissions portfolio | NA | NA | NA | Meeting minimum requirements (interview), admission not reported | According to rank and preference |

*Note:* UCAT = University Clinical Aptitude Test-Australia and New Zealand; ATAR = Australian Tertiary Admissions Rank; GPA = Grade Point Average; RA = Australian Statistical Geographical Classification-Remoteness Area; MMI = Multiple Mini-Interview; NA = Not applicable; MM = Modified Monash; FTE = Full-Time Equivalent; ISAT = International Student Admissions Test; WAM = Weighted Average Mark; conflicting information indicates that inconsistent information is reported on different parts of the website; citizenship information, prerequisite subjects, and English language requirements were omitted from the reporting of minimum entry requirements.
