# Supplementary material for: Medical school admission processes to target rural applicants: an international scoping review and mapping of Australian practices
Source: BMC Med Educ. 2025 May 6;25:659. doi: 10.1186/s12909-025-07234-3 (PMC12057111; doi:10.1186/s12909-025-07234-3)
Supplement: Supplementary file 5 — Supplementary Material 5 [file 12909_2025_7234_MOESM5_ESM.docx]

**Supplementary Table 5.** Summary of studies included in the scoping review

| **Study** | **Country** | **Details about the admissions process(es) investigated** | **Aims** | **Study design and type** | **Participants** | **Outcomes of interest** |
| --- | --- | --- | --- | --- | --- | --- |
| Ballejos et al., 2018 [1] | US | To facilitate rural physicians/representatives acting as interviewers, a video conference option was included. Virtual interviews were conducted the same as face-to-face and no additional training was provided. Interviews lasted 30-60 min and the final score was based on interest/suitability for a career in medicine, problem solving/communication skills, experiences, personal attributes, letters of recommendation, and a written application. | To evaluate the impact of a videoconference platform on rates of admission and to determine whether this process yielded a more diverse admitted sample than a face-to-face interview platform. | Quantitative – Review of administrative data | All applicants (N = 752) who interviewed for a place between 2014 and 2016 | Odds of acceptance for applicants from rural and non-rural high schools interviewed face-to-face and via video conference |
| Beattie et al., 2019 [2] | Australia | Selection is based on undergraduate GPA, GAMSAT, and MMI. Because of lower entry scores, bonuses are applied for rural residency. | To examine participants medical school selection data and academic performance in the Deakin University BMBS course between rural and metropolitan background students. | Quantitative survey | Medical school graduates (N = 147) | Performance of rural and metropolitan applicants on GPA, GAMSAT, and MMI |
| Curtis et al., 2017 [3] | New Zealand | There are separate pathways for indigenous and rural applicants, but applicants can only apply for one pathway. General entry and rural applicants are selected via prior academic performance (60%), interview score (25%), and UMAT score (15%). | To examine the association between admission variables and academic outcomes for students admitted into the medical programme under equity admission pathways in comparison to those students admitted under the general admission pathway. | Quantitative – Review of administrative data | Domestic students entering year two between 2002 and 2012 (N = 1676) | Admission GPA within the general, maori/pacific, and rural admission pathways |
| Dallaghan et al., 2021 [4] | US | The university established pipeline programs to recruit rural middle school, high school, and college students. | To present a model for analysing efforts for recruiting rural students, specifically, the Middle School Science and Technology Enrichment Program (middle school), Health Professions Recruitment and Exposure Program (high school), and Medical Education Development Program (college) | Quantitative – Review of administrative data | In-state applicants for the 2017-2019 admission cycles (N = 2791) | Proportion of rural and urban applicants who applied, were interviewed, and were admitted |
| Evans et al., 2020 [5] | US | Rurally aligned admissions processes across US medical schools were explored. | To examine the targeted admissions policies and practices of US allopathic and osteopathic medical schools. | Quantitative survey | US medical school representatives with positions such as deans/director/coordinator of admissions (N = 133) | Strategies used by US medical schools to target rural applicants |
| Fox et al., 2023 [6] | Australia | The university aims to interview an equal number of local (rural), rural, and metropolitan applicants. The MMI is completed online, comprising eight seven-minute stations with a regional focus for two stations. MMI contributes 50% to the admissions rank. | To explore how rural and metropolitan background applicants to a regionally focused training pathway perceive the MMI process, including differences in preparation and support leading up to the MMI. | Mixed-methods survey | Provisional entry applicants who participate in an MMI in 2022 (N = 295) | Rural and metropolitan applicant’s perception of a rurally aligned MMI for a provisional entry regional pathway |
| Gilbert et al., 2003 [7] | US | At the medical school, applicants are ranked according to undergraduate GPA and MCAT, with additional GPA points possible depending on selectivity of the undergraduate institution. The selectivity rating considers freshman test scores, freshman high school class rank, and factors such as college acceptance rates. | To determine if the practice of adjusting undergraduate grade point averages based on applicant undergraduate institution selectivity was detrimental to rural applicants. | Quantitative – Review of administrative data | In-state applicants between 1996 and 1999 who had sat the MCAT (N = 2033) | MCAT and GPA scores of rural and metropolitan applicants.  Proportion of rural and non-rural applicants who would have received an interview without selectivity adjustments, with adjustments, and if adjustments were doubled |
| Glasser et al., 2008 [8] | US | The Rural medical Education (RMED) program admits 15-20 students per year who pledge to return to practice primary care following residency. Students apply to the university and RMED program with RMED applicants assessed on academic merit and compatibility with the program. | To present the characteristics and results of the Rural Medical Education Program which addresses medical workforce needs focused on reducing rural health disparities. | Quantitative – Review of administrative data | Applicants from 1993-2007 (N = 18-85 per year) | Number of applicants, interviews, offers, and acceptances in the RMED program.  MCAT and cognitive characteristics of rural and nonrural students |
| Griffin et al., 2019 [9] | Australia | Re-testing on the UMAT was assessed via state-wide data. | To compare the profile of those who chose to reapply after initial failure with those who did not reapply and whether retesting gains were related to any demographic factors, thereby assessing the extent that re-application acts against effort to widen participation of under-represented groups in medicine. | Quantitative – Review of administrative data | Domestic applicants to undergraduate programs in New South Wales in 2013 and 2014, excluding international and indigenous applicants (N = 4007 unsuccessful in 2013, 3454 re-applicants/first time applicants in 2014) | Likelihood of reapplying after being unsuccessful.  Demographics which affect re-testing performance |
| Griffin et al., 2021 [10] | Australia | Undergraduate applicants in Australia and New Zealand in 2020 were the first to sit the UCAT, replacing the UMAT. Test scores are used to select for interview, out of a large number of applicants. | To identify whether the change in selection test for the 2020 intake was associated with changes in the influence of sex, socio-economic and remoteness of residence, and coaching on interviewee selection in NSW. | Quantitative – Review of administrative data | Applicants to three New South Wales undergraduate medical programs for 2019/2020 entry (N = 4114 [2019]; 4270 [2020]; 703 [applied to both intakes]) | UMAT and UCAT scores of rural and metropolitan applicants |
| Hay et al., 2017 [11] | Australia | Rural origin students admitted on lower scores are still above the 90^th^ percentile. Two large databases were merged to facilitate a multicentre analysis of selection and practice intentions. | To identify the relationship between selection scores and future practice intentions of rural origin medical students selected via a macro level reform designed to address the shortage of generalist medical practitioners in regional and rural Australia and to determine course performance of rural origin students selected on lower scores than their urban peers. | Quantitative – Review of administrative data | Undergraduate medical students commencing 2006 to 2013 across nine schools (aim 1); commencing medical students across 2006-2014 (single program)  N = 3573 [commencing questionnaire]; 981 [exit questionnaire]; 461 rural and 1431 urban students | Performance of rural and metropolitan applicants on UMAT, ATAR, and interview.  Relationship between admissions performance and rural practice intent |
| Henry, Edwards & Crotty, 2009 [12] | Australia | Evidence was gathered to guide the development of selection processes for a new medical school, focusing on selection processes and training experiences which promote the likelihood that students will later pursue a rural career. | To assess international and national best practice in the selection of students for graduate entry medical courses, to investigate correlations between medical student selection procedures and exposure to rural medical practice during medical training with choice of careers in rural medicine. | Qualitative interviews | Selection officers and key staff in Australian medical schools offering graduate entry programs in 2007 (N = 9 schools) | Strategies used to select rural applicants |
| Hutten-Czapski, Pitblado & Rourke, 2005 [13] | Canada | Admissions outcomes of Ontario medical schools were explored. | To determine whether rural Ontario applicants are less likely to apply to Ontario medical schools, and whether they have lower marks and are less likely to get into medical school. | Quantitative – Review of administrative data | Applicants to five Ontario medical schools in 2002 and 2003 (N = 4948) | GPA and MCAT performance and proportion admitted of rural and metropolitan background applicants |
| Langer et al., 2020 [14] | US | Interviews assess characteristics such as interpersonal and leadership skills. In 2018, the school transitioned from traditional to hybrid (traditional and MMI) interviews. | To assess whether evaluation metrics were comparable across key demographic variables following introduction of a new interview process and determine if the hybrid interview process was well-received by applicants. | Quantitative survey and analysis of administrative data | All applicants who interviewed during the 2019 admission cycle (N = 606; single program) | Performance of applicants from rural and non-rural counties on traditional interviews and MMIs |
| Larkins et al., 2015 [15] | International | The Training for Health Equity Network (THEnet) includes 11 medical schools with a social accountability mandate.  A key strategy is recruitment of students from underserved groups, more likely to return to their communities of origin. | To determine the kinds of selection strategies used by socially accountable medical schools, the extent that underserved populations are represented in these medical schools, and practice intentions of students at the time of selection. | Mixed-methods: review of institutional documentation, correspondence with senior staff, student survey | Students in one of five THEnet schools between 2012 and 2013; senior personnel from the medical schools (N = 944 surveys) | Admission strategies used in socially accountable medical schools, representation of underserved population, practice intent at selection |
| Lin et al., 2021 [16] | Taiwan | Interview eligibility is assessed via high school GPA, General Scholastic Ability Test, and a personal statement. Taiwanese medical schools use MMIs and non-MMI interview formats. MMI stations ranged from 5 to 10 across schools and the MMI accounted for 40-70% of the total score.  JCEWT is an alternative channel of entry used by all medical schools. JCEWT is a computerised standardised test assessing cognitive abilities. Candidates applying via the JCEWT were selected based on scores (ranking) and medical school priorities. | To compare the demographic characteristics and motivations for studying medicine of first-year medical students who were admitted to medical schools through either multiple mini-interview or the Joint College Entrance Written Test Channel. | Quantitative survey | First-year medical students from seven medical schools selected using MMI and JCEWT in 2016, excluding special entry pathways (N = 164) | Proportion of rural and metropolitan high school students accepted via MMI and JCEWT channels.  Relationship between high school location and likelihood of being accepted. |
| Longo, Gorman & Ge, 2005 [17] | US | The school considers rural status when making admissions decisions to help meet rural health care needs. | To determine whether the proportion of rural and nonrural students applying and accepted reflects the population. To assess whether MCAT and undergraduate GPA are similar for rural and urban applicants and whether any factors identify applicants more likely to be accepted. | Quantitative – Review of administrative data | Applicants between 1993 and 2000, excluding 1997 (missing data) (N = 667) | MCAT, GPA, and interview performance of rural and non-rural applicants and enrolled students |
| Matsumoto, Inoue & Kajii, 2008 [18] | Japan | All students sign contracts that they are fully funded by their home prefectures for six years of undergraduate education and commit to working in their home prefectures for 9 years after graduation. Students who breach the obligation must pay all medical school expenses. | To describe the personal, familial and academic characteristics of rural and urban students and their career choices and to determine correlations between geographic/demographic indicators of places of origin and future workplaces. | Quantitative – survey and analysis of administrative data | Students who had completed their 9-year return of service obligation by 2006 (single program)  N = 1929 [baseline data]; 98.7% response rate [2009], 98.2% 2002, 98% 2006 | Academic standing at entry under a rurally focussed admissions process |
| Ozeki et al., 2022 [19] | Japan | A regional quota was incorporated into the admissions system in 2008. General admission includes early and second rounds and the regional quota includes AO-EXAM and REC-EXAM. Regional quota applicants come from areas close to the school and intend to complete postgraduate training and practice in the local communities after graduation. All applicants take the National Center Test. | To determine whether the academic performance of medical students differs based on admission types and to explore its predictors by analysing a decade's worth of admissions data. Also aims to investigate the extent to which university-specific subject tests influence academic success during medical school. | Quantitative – Review of administrative data | Students commencing 2010 through 2019 (N = 1057; single program) | National Center Test and university specific examination performance and high school GPA of students admitted via the rural and general pathways |
| Pang et al., 2021 [20] | Malaysia | MMIs developed included three interviewer-led, scenario-driven stations (motivation and preparation, ethics dilemma, professionalism/teamwork) along with two role player-led, observer-assessed stations (breaking bad news/empathy, science-communication/performing simple mathematics under duress). Each station assessed two domains including motivation/preparation, teamwork/leadership, ethics, professionalism, empathy, communication, and logical thinking and general impressions. Novel aspects of this MMI included fourth-years medical students as role players and that candidates could answer in English or the national language. | Not reported | Quantitative – Review of administrative data | Candidates who sat the MMI in 2019 (N = 260) | MMI performance of urban and semi-urban/rural applicants |
| Puddey & Mercer, 2013 [21] | Australia | Evaluation of the UMAT | To investigate associations between socioeconomic advantage/disadvantage with each UMAT section and overall in relation to other demographic predictors. Performance in relation to secondary school, rural background and Aboriginal/Torres Strait Islander self-identification is also reported on. | Quantitative – Review of administrative data | Australian applicants who sat the UMAT between 2000 and 2012 for the first time (N = 118,085) | UMAT performance for each subtest and overall for applicants of differing rurality |
| Puddey & Mercer, 2014 [22] | Australia | In 2004, the first round of selection was undertaken for graduate entry into the medical course. Interview shortlisting is via undergraduate GPA and GAMSAT scores (equally weighted) and the interview is highly structured, focussing on communication. GPA, GAMSAT, and interview are equally weighted to determine admission offers or students can be selected via an alternate rural entry pathway utilising the same selection elements. | To determine the relationship between the students' entry scores, demographic characteristics and background discipline with subsequent performance in the course. | Quantitative – Review of administrative data | Graduate entry students (excluding indigenous) between 2005 and 2012 (N = 421) | Association between rural entry and GPA, GAMSAT, and interview performance for all pathways excluding indigenous |
| Puddey et al., 2011 [23] | Australia | Revised selection processes were introduced in 1999 to increase diversity and improve equity. Revised processes include a structured interview, and prior academic performance (previously the sole admission criteria). | To evaluate the possible further influence of either the structured interview or UMAT and analyse the relationship of each of the selection components with the demographic composition of student cohorts both before and after commencement of the revised selection processes. | Quantitative – Review of administrative data | All standard entry (including rural special entry) students between 1985 to 2011 (N = 2839) | Performance of applicants admitted via the general entry (academic performance only) and rural special entry pathways (academic performance, interview, and UMAT) |
| Puddey et al., 2014 [24] | Australia | For urban applicants, ATAR, interview and UMAT are weighted at 2:2:1 (previously 1:1:1) and for rural applicants, rurality, ATAR, interview and UMAT are weighted at 1:1.2:1.2:0.6. The proportion of rural students admitted has risen from 20% in 2006 to 29% in 2011. | To analyse intended career destinations of commencing medical students against the criteria utilised for their selection into medical school, together with socio-demographic factors that could potentially influence rural versus urban practice intention. | Quantitative survey and review of administrative data | High school students who entered via the standard pathway between 2006 and 2011 and responded to the survey (N = 667) | ATAR, UMAT, and interview scores of rural and metropolitan background applicants |
| Puddey et al., 2015 [25] | Australia | In 1999, a revised entry process was introduced, including interview and UMAT as well as ATAR (previously used in isolation). | To analyse the demographic profile of entering medical students together with those factors used in their selection for medical school, as potential predictors of those who ultimately have elected to practice in a rural site. | Quantitative – Review of administrative data | Standard entry applicants from 1999 to 2006 (N = 729) | ATAR, UMAT, and interview performance and practice location of rural and urban background students |
| Raghavan et al., 2011 [26] | Canada | Documented the process of revising admissions processes to increase the volume of offers extended to rural applicants. | To report on the methodology adopted at the faculty to bring about the changes in admissions procedures. | Mixed-methods: literature review/environmental scan, priority matrix, survey, mathematical modelling/analysis of administrative data | Applicants between 2000 and 2007 (retrospective; N = 2041) and 2009 (prospective; N = 327) | Proportion of applicants admitted from rural and urban high schools after the implementation of revised rurally aligned admission processes |
| Raghavan et al., 2013 [27] | Canada | For 10 of the 11 stations, applicants had 2-minutes to read the question or scenario and eight-minutes to respond to the single interviewer. The final station was a 10-minute writing station. Stations were scored out of 7. | To determine if there is an association between MMI scores and high school location (rural or urban), rural connections, employment in rural areas, and rural community services. | Quantitative – Review of administrative data | Applicants who interviewed for admission between 2008 and 2011 (N = 1257) | MMI, MCAT, and GPA performance of rural and non-rural applicants.  Correlation between MMI and GPA and MCAT |
| Ray, Woolley & Sen Gupta, 2015 [28] | Australia | Selection combines rurally adjusted academic achievement, a written personal statement demonstrating attributes relevant to rural medical practice, and panel interview scores. | To determine the impact of JCU medical school's policy of preferentially selecting rural and remote background students on undergraduate performance across the 6 years of the course and on graduate practice location. | Quantitative – Review of administrative data | Domestic students accepted between 2000 and 2008 (N = 804) | Rurally focussed admissions process - Tertiary entrance/OP, and interview scores for RA1, 2, 3, and 4 or 5 applicants.  Tertiary entrance/OP and interview score for those who accepted a CSP and BMP |
| Schmitz et al., 2020 [29] | US | Examined rurally aligned admissions processes in US medical schools generally. | To examine the characteristics and practices of U.S. allopathic and osteopathic medical schools reporting a rurally targeted admissions approach, including their motivations, resources, and challenges. | Mixed-methods: Survey and semi-structured interviews | US medical school employees involved in designing admission policies (N = 133; survey).  Survey respondents from medical school targeting applicants likely to practice in rural areas and with rurally aligned mission on their website (N = 10; interview). | Information about rural recruitment and selection processes |
| Turnbull et al., 2003 [30] | Australia | Selection is based on UMAT scores, a 45-minute structured interview and a tertiary entrance rank of ≥90%. Admission was previously based on solely academic performance but was revised based on research relating to motivation to study medicine as well as equity concerns raised and national policy recommendations. | To report on four case studies into various aspects of the selection process | Mixed-methods: Multiple case studies | Medical school applicants between 1994 and 2000. | Psychosocial and sociodemographic characteristics and attrition rates of selected students |
| Upadhyay et al., 2017 [31] | Nepal | Applicants were invited for interview based on their mental agility test (MAT-score). Interview was an Objective Structured Performance Examination (similar to MMI). Preferential credits are allocated based on sociodemographic characteristics including rurality. Admissions decisions were based on OSPE scores and social inclusion matrix (preferential credits) for scholarship-seeking applicants and a personal quality assessment and OSPE for fee-paying students. Scholarship students agree to serve for 2-4 years in a rural or remote area. | To describe the innovative medical student selection scheme adopted by Patan Academy of Health Sciences for its Bachelor of Medicine and Bachelor of Surgery (MBBS) program and its initial findings. | Quantitative – Review of administrative data | Students enrolled from 2010 to 2012 (N = 175) | Proportion of students admitted with and without rural characteristics (geographical region and rural work experience) |
| Wright & Woloschuk, 2008 [32] | Canada | High school graduates are assessed based on GPA and MCAT. Each applicant’s file is reviewed by two members of the selection committee and if shortlisted for interview, six reviewers assess the application and score it out of 5. The average of the reviewer scores is used to rank applicants for admission. | To determine how successfully rural background applicants, compared with regional and urban background applicants, progressed past the interview stage to admission. | Quantitative – Review of administrative data | Alberta residents who applied from 1991 to 2000 (N = 4407) | GPA, MCAT, and reviewer scores of urban, regional, and rural applicants.  Proportion or urban, regional, and rural Albertans interviewed and admitted |

*Note:* US = United States; GPA = Grade Point Average; GAMSAT = Graduate Medical School Admissions Test; MMI = Multiple Mini-Interview; BMBS = Bachelor of Medicine Bachelor of Surgery; UMAT = Undergraduate Medicine and Health Sciences Admissions Test; UCAT = University Clinical Aptitude Test; MCAT = Medical College Admissions Test; ATAR = Australian Tertiary Admissions Rank; JCEWT = Joint College Entrance Written Test; AO-EXAM = admissions office exam (self-referral); REC-EXAM = recommendation examination (recommended by the high school); BMP = Bonded Medical Place; CSP = Commonwealth Supported Place.

**References**

1. Ballejos MP, Oglesbee S, Hettema J, Sapien R. An equivalence study of interview platform: Does videoconference technology impact medical school acceptance rates of different groups? Adv Health Sci Educ. 2018;23:601-10.

2. Beattie J, D'Souza K, Mc Leod J, Versace V. Rural origin students match metropolitan origin students' academic performance once admitted to Bachelor of Medicine Bachelor of Surgery course. Aust J Rural Health. 2019;27:181-2.

3. Curtis E, Wikaire E, Jiang Y, McMillan L, Loto R, Poole P, et al. Examining the predictors of academic outcomes for indigenous Māori, Pacific and rural students admitted into medicine via two equity pathways: A retrospective observational study at the University of Auckland, Aotearoa New Zealand. BMJ Open. 2017;7:e017276.

4. Dallaghan GLB, Spero JC, Byerley JS, Rahangdale L, Fraher EP, Steiner B. Efforts to recruit medical students from rural counties: A model to evaluate recruitment efforts. Cureus J Med Sci. 2021;13: e17464.

5. Evans DV, Jopson AD, Andrilla CHA, Longenecker RL, Patterson DG. Targeted medical school admissions: A strategic process for meeting our social mission. Fam Med. 2020;52:474-82.

6. Fox JL, Batacan R, Saluja S, Pullen C, McGrail M. Experiences of rural and metropolitan background applicants in preparing for and completing a regionally focused multiple mini-interview for admission into a regional medical program. Educ Health. 2023;36:116-22.

7. Gilbert GE, Blue AV, Basco WT. The effect of undergraduate GPA selectivity adjustment on pre-interview ranking of rural medical school applicants. J Rural Health. 2003;19:101-4.

8. Glasser M, Hunsaker M, Sweet K, MacDowell M, Meurer M. A comprehensive medical education program response to rural primary care needs. Acad Med. 2008;83:952-61.

9. Griffin B, Auton J, Duvivier R, Shulruf B, Hu W. Applicants to medical school: If at first they don't succeed, who tries again and are they successful? Adv Health Sci Educ. 2019;24:33-43.

10. Griffin B, Horton GL, Lampe L, Shulruf B, Hu W. The change from UMAT to UCAT for undergraduate medical school applicants: Impact on selection outcomes. Med J Aust. 2021;214:84-9.

11. Hay M, Mercer AM, Lichtwark I, Tran S, Hodgson WC, Aretz HT, et al. Selecting for a sustainable workforce to meet the future healthcare needs of rural communities in Australia. Adv Health Sci Educ. 2017;22:533-51.

12. Henry JA, Edwards BJ, Crotty B. Why do medical graduates choose rural careers? Rural Remote Health. 2009;9:1083.

13. Hutten-Czapski P, Pitblado R, Rourke J. Who gets into medical school? Comparison of students from rural and urban backgrounds. Can Fam Physician. 2005;51:1240-1.

14. Langer T, Ruiz C, Tsai P, Adams U, Powierza C, Vijay A, et al. Transition to multiple mini interview (MMI) interviewing for medical school admissions. Perspect Med Educ. 2020;9:229-35.

15. Larkins S, Michielsen K, Iputo J, Elsanousi S, Mammen M, Graves L, et al. Impact of selection strategies on representation of underserved populations and intention to practise: International findings. Med Educ. 2015;49(1):60-72.

16. Lin CH, Chen MH, Tsai TC, Huang WJ. Difference in demographics and motivation to study medicine with respect to medical students' channel of admission: A national study. Med Teach. 2021;43:1025-30.

17. Longo DR, Gorman RJ, Ge B. Rural medical school applicants: Do their academic credentials and admission decisions differ from those of nonrural applicants? J Rural Health. 2005;21:346-50.

18. Matsumoto M, Inoue K, Kajii E. Characteristics of medical students with rural origin: Implications for selective admission policies. Health Policy. 2008;87:194-202.

19. Ozeki S, Kasamo S, Inoue H, Matsumoto S. Does regional quota status affect the performance of undergraduate medical students in Japan? A 10-year analysis. Int J Med Educ. 2022;13:307-14.

20. Pang N, Kadir F, Kamu A, Mun HC, Loo JL, Ahmedy F, et al. A pilot project to introduce the multiple mini interview (MMI) at a Borneo medical school: The universiti Malaysia Sabah experience- A cross-sectional study. Ann Med Surg. 2021;71.

21. Puddey IB, Mercer A. Socio-economic predictors of performance in the Undergraduate Medicine and Health Sciences Admission Test (UMAT). BMC Med Educ. 2013;13:155.

22. Puddey IB, Mercer A. Predicting academic outcomes in an Australian graduate entry medical programme. BMC Med Educ. 2014;14:31.

23. Puddey IB, Mercer A, Carr SE, Louden W. Potential influence of selection criteria on the demographic composition of students in an Australian medical school. BMC Med Educ. 2011;11:97.

24. Puddey IB, Mercer A, Playford DE, Pougnault S, Riley GJ. Medical student selection criteria as predictors of intended rural practice following graduation. BMC Med Educ. 2014;14:218.

25. Puddey IB, Mercer A, Playford DE, Riley GJ. Medical student selection criteria and socio-demographic factors as predictors of ultimately working rurally after graduation. BMC Med Educ. 2015;15.

26. Raghavan M, Martin BD, Roberts D, Aoki F, MacKalski BA, Sandham JD. Increasing the enrolment of rural applicants to the faculty of medicine and addressing diversity by using a priority matrix approach to assign values to rural attributes. Rural Remote Health. 2011;11:1646.

27. Raghavan M, Martin BD, Burnett M, Aoki F, Christensen H, Mackalski B, et al. Multiple mini-interview scores of medical school applicants with and without rural attributes. Rural Remote Health. 2013;13:2362.

28. Ray RA, Woolley T, Sen Gupta T. James Cook University's rurally orientated medical school selection process: Quality graduates and positive workforce outcomes. Rural Remote Health. 2015;15:3424.

29. Schmitz DF, Evans DV, Andrilla CHA, Jopson AD, Longenecker RL, Patterson DG. Challenges and best practices for implementing rurally targeted admissions in u.S. medical schools. J Health Care Poor Underserved. 2020;31:320-31.

30. Turnbull D, Buckley P, Robinson JS, Mather G, Leahy C, Marley J. Increasing the evidence base for selection for undergraduate medicine: Four case studies investigating process and interim outcomes. Med Educ. 2003;37:1115-20.

31. Upadhyay SK, Bhandary S, Bhandari DB, Dulal RK, Baral KP, Gongal RN, et al. Admitting deserving medical students from rural and disadvantaged: Patan Academy of Health Sciences' approach. J Nepal Health Res Counc. 2017;15:75-80.

32. Wright B, Woloschuk W. Have rural background students been disadvantaged by the medical school admission process? Med Educ. 2008;42:476-9.
