# Supplementary material for: Medical school admission processes to target rural applicants: an international scoping review and mapping of Australian practices
Source: BMC Med Educ. 2025 May 6;25:659. doi: 10.1186/s12909-025-07234-3 (PMC12057111; doi:10.1186/s12909-025-07234-3)
Supplement: Supplementary file 6 — Supplementary Material 6 [file 12909_2025_7234_MOESM6_ESM.docx]

**Supplementary Table 6.** Quantitative results from studies included in the scoping review

| **Study** | **Key findings** |
| --- | --- |
| Ballejos et al., 2018 [1] | Applicants interviewed face-to-face were older (25.6 ± 4.7 years) than those interviewed virtually (24.4 ± 2.7 years; *P* = 0.01).  Of accepted applicants, those who interviewed face-to-face were older (24.0 ± 2.3 vs 25.4 ± 3.9 years; *P* = 0.04) and had a lower GPA (3.76 ± 0.18 vs 3.65 ± 0.25; *P* = 0.02).  Those interviewed online were less likely to be accepted than those interviewed face-to-face (aOR = 0.89; CI: 0.55 – 1.45).  High school rurality was not associated with acceptance; however, of face-to-face interviewees, urban (OR = 1.81; CI: 1.17 – 2.79) and non-urban (OR = 1.80; CI = 1.01 – 3.21) applicants were more likely to be accepted than those from out of state. |
| Beattie et al., 2019 [2] | GPA was lower in rural (mean 6.45) compared to metropolitan (6.61) applicants (*P* = 0.021).  GAMSAT scores were similar in rural (61.27) and metropolitan (62.51) applicants (*P* = 0.23).  MMI scores were similar in rural (78.12) and metropolitan (78.88) applicants (*P* = 0.48). |
| Curtis et al., 2017 [3] | GPA was significantly lower for those admitted via the rural entry pathway (7.72 ± 0.88) compared to general entry (8.12 ± 0.99; *P* = 0.0002). |
| Dallaghan et al., 2021 [4] | Of 257 rural applicants, 130 (51%) were interviewed and 54 (42% interviewees) were admitted.  Of 2534 urban applicants, 1268 (50%) were interviewed and 498 (39% interviewees) were admitted. |
| Evans et al., 2020 [5] | Public medical schools (N = 60; 79%) were more likely to report rurally targeted admissions processes than private institutions (N = 32; 56.1%; P <0.01).  Medical schools conducted career exploration/counselling/mentorship in high schools (N = 75; 83.3%), area health education center (AHEC) programs (N = 54; 66.7%), community/technical colleges (N = 54; 62.1%), 4-year universities (N = 80; 89.9%), and postbaccalaureate (pb) programs (N = 60; 68.2%).  Medical schools completed academic enhancement activities in high schools (n = 24; 28.2%), AHEC programs (N = 20; 24.7%), community/technical colleges (N = 19; 22.4%), 4-year universities (N = 41; 47.7%), and pb programs (N = 40; 45.5%).  Admissions preparation activities were conducted in community/technical colleges (N = 30; 35.7%), 4-year universities (N = 50; 57.5%), pb programs (N = 41; 46.6%).  Articulation agreements were used in community/technical colleges (N = 6; 7.2%), 4-year universities (N = 36; 42.9%), and pb programs (N = 17; 20.5%).  Characteristics used to identify students likely to practice rurally included graduating from a rural high school (N = 69; 76.7%), growing up in a rural community (N = 89; 97.8%), volunteering in a rural community (N = 72; 80.9%), being previously employed in a rural community (N = 56; 66.7%), having a partner/spouse receptive to living in a rural community (N = 10; 13.7%), graduating from a public university (N = 27; 32.1%), having positive rural exposure (N = 45; 52.9%), interest in family medicine (N = 61; 70.1%), having a non-continuous path from high school (N = 37; 43.0%), and from a group underrepresented in medicine (N = 69; 79.3%).  Selection strategies used at the time of application to select students likely to enter rural practice included secondary application questions (N = 65; 74.7%), targeted financial aid (N = 33; 37.1%), modified MCAT cutoffs (N = 18; 21.45), reserved places in each cohort (N = 18; 20.2%), and modified GPA cutoffs (N = 16; 18.8%).  Selection strategies used during interview to select students likely to enter rural practice included rural physicians as interviewers (N = 65; 75.6%), preferential scoring during interview screening (N = 34; 38.2%), preferential scoring in final admissions determination (N = 27; 30%), undergoing other admissions processes (N = 13; 14.6%), separate interview to other applicants (N = 10; 11.2%), and different interview questions (N = 6; 6.8%).  When selecting students likely to enter rural practice, key personnel involved included the dean of admissions (N = 76; 89.4%), physicians practicing in the specific area (N = 59; 72%), and the director of the pathway (N = 46; 61.3%). |
| Fox et al., 2023 [6] | Rural (N = 36; 68%) and metropolitan applicants (N = 17; 56.7%) mostly agreed that the MMI is fair to all applicants, allowed applicants to demonstrate their strengths (N = 27; 58.7% rural vs 17; 54.8% metropolitan), aligned with expectations (N = 38; 79.2% rural vs 28; 87.5% metropolitan), and helps a rural program select the most suitable applicants (N = 30; 62.5% rural vs 17; 58.6% metropolitan; all *P* >0.05).  Rural applicants were more likely to agree that the MMI gave them an advantage over other applicants (N = 30; 61.2%) compared to metropolitan applicants (N = 7; 22.6%; *P* <0.05).  Most rural and metropolitan applicants felt that the MMI should contribute <50% (N = 22 [45.8%] rural vs 12 [40%] metropolitan) or 50% (N = 20 [41.7%] rural vs 15 [50%] metropolitan; *P* >0.05). |
| Gilbert et al., 2003 [7] | Mean MCAT biological science scores were higher for rural (8.9) than nonrural (8.5) applicants in 1996 (P >0.0025) but lower in 1997 (7.3 vs 8.5), 1998 (7.2 vs 8.4), and 1999 (7.8 vs 8.7) (*P* <0.0025 in two years).  Rural applicants had lower MCAT physical science scores in 1996 (8.3 vs 8.5; *P* >0.0025), 1997 (6.7 vs 8.1; *P* <0.0025), 1998 (6.7 vs 8.2; *P* <0.0025), and 1999 (7.3 vs 8.2; *P* <0.0025).  MCAT verbal reasoning scores were similar for rural and nonrural applicants in 1996 (8.5 rural vs 8.8 nonrural), 1997 (8.0 vs 8.7), 1998 (7.8 vs 8.6), and 1999 (8.0 vs 8.7; all *P* >0.0025).  Mean unadjusted GPA was similar for rural and nonrural applicants in 1996 (both 3.31), 1997 (3.26 vs 3.35), 1998 (3.34 vs 3.32), and 1999 (3.38 vs 3.36; all *P* >0.0025).  Mean adjusted GPA was similar for rural and nonrural applicants in 1996 (3.59 vs 3.68), 1997 (3.51 vs 3.69), 1998 (3.62 vs 3.67), and 1999 (3.66 vs 3.72; all *P* >0.0025).  Without selectivity adjustments, 21 (63.6%) rural and 299 (84.4%) non rural applicants would have received an interview in 1996, 20 (40.8%) rural and 319 (69.7%) nonrural in 1997, 28 (57.1%) rural and 320 (68.8%) nonrural in 1998, and 28 (49.1%) rural and 325 (57.2%) nonrural in 1999.  With selectivity adjustments, 20 (60.6%) rural and 300 (85.4%) non rural applicants would have received an interview in 1996, 21 (44.9%) rural and 318 (69.4%) in 1997, 27 (55.1%) rural and 321 (69%) nonrural in 1998, and 28 (49.1%) rural and 325 (57.2%) nonrural in 1999.  With selectivity adjustments doubled, 20 (60.6%) rural and 300 (84.8%) nonrural applicants would have received an interview in 1996, 24 (49%) rural and 314 (68.6%) nonrural in 1997, 28 (57.1%) rural and 320 (68.8%) nonrural in 1998, and 25 (43.9%) rural and 357 (62.9%) nonrural applicants in 1999. |
| Glasser et al., 2008 [8] | In 1993 there were 18 applications, 12 applicants interviewed, 6 offered a rural place, and 5 admitted to the university with a rural place.  In 1994 there were 46 applications, 25 interviewed, 20 offered a rural place, and 17 admitted to the university with a rural place.  In 1995 there were 59 applications, 34 interviewed, 20 offered a rural place, and 17 admitted to the university with a rural place.  In 1996 there were 84 applications, 31 interviewed, 20 offered a rural place, and 20 admitted to the university with a rural place.  In 1997 there were 84 applications, 31 interviewed, 23 offered a rural place, and 16 admitted to the university with a rural place.  In 1998 there were 75 applications, 32 interviews, 22 offered a rural place, and 17 admitted to the university with a rural place.  In 1999 there were 62 applications, 32 interviewed, 25 offered a rural place, and 20 admitted to the university with a rural place.  In 2000 there were 54 applications, 31 interviewed, 23 offered a rural place, and 16 admitted to the university with a rural place.  In 2001 there were 76 applications, 32 interviewed, 23 offered a rural place, and 18 admitted to the university, 16 with a rural place.  In 2002 there were 85 applications, 30 interviewed, 16 offered a rural place, and 16 admitted to the university with a rural place.  In 2003 there were 51 applications, 22 interviewed, 20 offered a rural place, and 20 admitted to the university, 15 with a rural place.  In 2004 there were 32 applications, 25 interviewed, 22 offered a rural place, and 20 admitted to the university, 11 with a rural place.  In 2005 there were 38 applications, 28 interviewed, 23 offered a rural place, and 21 admitted to the university, 14 with a rural place.  In 2006 there were 30 applications, 22 interviewed, 17 offered a rural place, and 17 admitted to the university, 14 with a rural place.  In 2007 there were 39 applications, 29 interviewed, 22 offered a rural place, and 21 admitted to the university, 14 with a rural place.  In class 1, MCAT scores (8.13 rural vs 9.35 nonrural) and cognitive characteristics (61.66 rural vs 65.03 nonrural) were lower in rural pathway students.  In class 2, MCAT scores (8.83 rural vs 9.13 nonrural) and cognitive characteristics (63.9 rural vs 64.6 nonrural) were lower in rural pathway students.  In class 3, MCAT scores (7.78 rural vs 9.18 nonrural) and cognitive characteristics (62.7 rural vs 65.2 nonrural) were lower in rural pathway students.  In class 4, MCAT scores (8.07 rural vs 8.9 nonrural) and cognitive characteristics (62.55 rural vs 64.5 nonrural) were lower in rural pathway students. |
| Griffin et al., 2019 [9] | Metropolitan applicants were more likely to re-apply (20.5%) than rural applicants (8%; *P* <0.001).  Re-applicants had a higher ATAR (92.4 ± 20.5 vs 74.8 ± 39.7; *P* <0.001), UMAT 1 scores (53.6 ± 8.6 vs 52.4 ± 9.7; *P* <0.01), and UMAT 3 scores (55.7 ± 8.5 vs 53.8 ± 9.8; *P* <0.01). UMAT 2 scores were similar (52.8 ± 8.9 vs 52.2 ± 9.5; *P* >0.05).  Re-applicants (25.4%) were more likely to be shortlisted for interview on their first attempt than those who did not re-apply (14.3%). |
| Griffin et al., 2021 [10] | UMAT 1 (50.4 ± 9.2 rural vs 55.2 ± 9.8 metropolitan), UMAT 2 (52.0 ± 9.7 vs 54.3 ± 9.1), UMAT 3 (51.1 ± 9.3 vs 56.3 ± 9.6), and total UMAT score (153.4 ± 12.2 vs 165.6 ± 22.7) were lower in rural applicants (all *P* <0.001).  UCAT verbal reasoning (851.5 ± 76.5 rural vs 604.1 ± 75.6 metropolitan), UCAT decision making (634.9 ± 72.5 vs 656.5 ± 78.4), UCAT quantitative reasoning (661.7 ± 75.4 vs 705.4 ± 86.2), UCAT abstract reasoning (640.3 ± 85.6 vs 676.6 ± 94.8), UCAT total score (2518 ± 234.3 vs 2643 ± 263.2), and UCAT situational judgement (610.9 ± 62.6 vs 623.2 ± 60.7) were lower in rural applicants (all *P* <0.001). |
| Hay et al., 2017 [11] | Rural applicants had lower ATAR (96.41 ± 3.08 vs 98.77 ± 1.59; *P* <0.001), interview scores (81.99 ± 11.02 vs 84.02 ± 9.78%; *P* = 0.001), and UMAT 1 (59.06 ± 7.19 vs 64.95 ± 8.42; *P* <0.001), 2 (56.83 ± 6.86 vs 59.49 ± 7.21; *P* <0.001), and 3 scores (60.4 ± 7.81 vs 67.79 ± 9.26; *P* <0.001) compared to urban applicants. |
| Hutten-Czapski, Pitblado & Rourke, 2005 [12] | 7.3% of applicants were rural origin and mean GPA for both rural and urban applicants was 3.42 (*P* = 0.995).  One in 5.6 rural and one in 4.7 urban applicants were admitted and the likelihood of being admitted was similar (*P* = 0.139). |
| Langer et al., 2020 [13] | Traditional interview scores were similar for those from rural and nonrural counties (group mean 3.89; *P* = 0.577).  MMI scores were similar for those from rural and nonrural counties (mean and P value not reported). |
| Larkins et al., 2015 [14] | The number and proportion of medical students who had spent the majority of primary school in a rural and regional town was 102 (62.6%) at JCU (Australia), 195 (91.1%) at WSU (South Africa), 87 (47.8%) at Gezira (Sudan), 175 (82.2%) at Ghent (Belgium), 11 (24.4%) at ADZU (Philippines), and 960 (31.6%) in comparative data from across Australia.  The number and proportion of medical students intending to practice with rural and remote populations was 86 (53.1%) at JCU, 130 (62.8%) at WSU, 54 (33.5%) at Gezira, and 29 (65.9%) at ADZU.  The number and proportion of students intending to practice in a small/remote village was 41 (25.3%) at JCU, 16 (7.5%) at WSU, 17 (10.4%) at Gezira, 0 at Ghent, 3 (6.7%) at ADZU, and 105 (3.1%) in comparative data from Australia and 0 in comparative data from Flanders (Belgium).  The number and proportion of students intending to practice in a small rural town was 24 (14.9%) at JCU, 77 (36.3%) at WSU, 14 (8.1%) at Gezira, 19 (15.3%) at Ghent, 14 (31.1%) at ADZU, and 176 (5.2%) in comparative data from Australia and 91 (26.35) in comparative data from Flanders.  The number and proportion of students intending to practice in a regional centre was 46 (28.4%) at JCU, 59 (27.8%) at WSU, 21 (12.1%) at Gezira, 46 (37.1%) at Ghent, 13 (28.9%) at ADZU, and 351 (10.4%) in comparative data from Australia and 105 (30.3%) in comparative data from Flanders. |
| Lin et al., 2021 [15] | 164 (80.4%) in the MMI group were from metropolitan high schools and 163 (86.2%) in the JCEWT were from metropolitan high schools.  Being from a metropolitan high school was not associated with being accepted via the MMI channel (*P* = 0.271). |
| Longo, Gorman & Ge, 2005 [16] | Biological science MCAT mean scores were lower in rural (8.6) than nonrural (8.9; *P* = 0.046) applicants and in rural applicants who were accepted (9.6) compared to nonrural applicants who were accepted (9.8; *P* = 0.0495).  Physical science MCAT scores were lower in rural (8.4) than nonrural (8.6; *P* = 0.0118) applicants and lower in rural applicants who were accepted (9.3) compared to nonrural applicants who were accepted (9.6; *P* = 0.0469).  Verbal MCAT scores were similar in rural and metropolitan applicants (8.8 vs 8.7; *P* = 0.5087) and rural and metropolitan applicants who were accepted (9.6 vs 9.6; *P* = 0.7852).  Total MCAT scores were similar for rural and nonrural applicants (25.8 vs 26.2; P = 0.1077) and rural and nonrural applicants who were accepted (28.5 vs 29.0; *P* = 0.3244).  Cumulative GPA was higher in rural than nonrural applicants (3.5 vs 3.4; *P* <0.0001) and higher in rural than nonrural applicants who were accepted (3.7 vs 3.6; *P* <0.0001).  Cumulative science GPA was higher in rural than nonrural applicants (3.4 vs 3.3; *P* <0.0001) and in rural than nonrural applicants who were accepted (3.7 vs 3.6; *P* = 0.0002).  Interview scores were similar for rural and nonrural applicants (25.9 vs 25.8; *P* = 0.3567) and rural and nonrural applicants who were accepted (both 27.6; *P* = 0.556).  Of accepted applicants, 35.2% were rural compared to 23.3% of non-accepted applicants (*P* <0.0001).  Rurality was associated with acceptance to medical school (OR = 2.094 [CI = 1.551 – 2.827]; P <0.0001). |
| Matsumoto, Inoue & Kajii, 2008 [17] | Academic standing at entrance was similar for rural (19.1 ± 0.8) and urban (19.0 ± 0.7) applicants (*P* = 0.15). |
| Ozeki et al., 2022 [18] | NCT early (83.52 ± 3.22) and later-EXAM (85.57 ± 3.01) were significantly higher than AO (81.61 ± 3.93) and REC-EXAM (80.65 ± 3.61; *P* <0.0001). |
| Pang et al., 2021 [19] | In station 1, rural and metropolitan applicants had similar scores for motivation (4.03 vs 4.07), teamwork (both 3.83), general impressions (3.97 vs 3.92), and overall (11.35 vs 11.82).  In station 2, rural applicants had lower scores for ethics (4.09 vs 4.18; P = 0.010) and similar scores for communication (4.07 vs 4.21), general impressions (4.13 vs 4.24), and overall (11.71 vs 12.26).  In station 3, rural applicants had lower scores for professionalism (4.09 vs 4.29; P = 0.011) and similar scores for teamwork (4.01 vs 4.26), general impressions (4.02 vs 4.04), and overall (11.54 vs 12.6).  In station 4, rural applicants had similar scores for empathy (3.18 vs 3.33), communication (3.39 vs 3.49), general impressions (3.42 vs 3.44), and overall (9.99 vs 10.24).  In stations 5, rural applicants had similar scores for logical thinking (3.59 vs 3.67), communication (3.84 vs 3.82), general impressions (3.78 vs 3.69), and overall (11.2 vs 11.18). |
| Puddey & Mercer, 2013 [20] | 93.4% of the sample were from schools defined as highly accessible (ARIA classification), 4.9% accessible, 1.2% moderately accessible, and 0.5% remote/very remote.  Compared to highly accessible areas, UMAT 1 scores were higher for those in accessible areas (*P* <0.01).  Compared to highly accessible areas, UMAT 2 scores were higher for those in accessible (*P* <0.0001), moderately accessible (P <0.01), or remote areas (*P* <0.05).  Compared to highly accessible areas, UMAT 3 scores were progressively lower (all *P* <0.001). |
| Puddey & Mercer, 2014 [21] | Entering via the rural pathway was associated with a lower GPA at entry (r = -0.165; *P* = 0.001), total GAMSAT (r = -0.120; *P* = 0.014), GAMSAT 3 (r = -0.157; *P* = 0.001), and interview scores (r = 0.124; *P* = 0.011). There was no significant association between entering via the rural pathway and GAMSAT 1 (r = 0.0002; *P* = 0.965) or 2 scores (r = 0.007; *P* = 0.885). |
| Puddey et al., 2011 [22] | Under the original admissions process, tertiary entrance scores were lower for those in the rural pathway (408.9 ± 2.6 vs 456.8 ± 0.8; *P* <0.001).  Under the revised admissions processes, tertiary entrance rank was lower for those in the rural pathway (97.71 ± 0.09 vs 99 ± 0.03; *P* <0.001).  Those in the rural pathway performed worse across all admission elements (all *P* <0.001 except UMAT 2 [*P* <0.01]). |
| Puddey et al., 2014 [23] | Rural applicants had a lower ATAR (97.9 ± 0.11 vs 99.1 ± 0.04; *P* <0.001), interview scores (25.5 ± 0.4 vs 27.0 ± 0.3; *P* = 0.004), overall UMAT percentile (76.2 ± 1.3 vs 89.8 ± 0.4; *P* <0.001), UMAT 1 percentile (77.3 ± 1.6 vs 86 ± 0.6; *P* <0.001), UMAT 2 percentile (74 ± 1.6 vs 78.3 ± 1; *P* = 0.028), and UMAT 3 percentile (66.1 ± 1.9 vs 85 ± 1.9; *P* <0.001). |
| Puddey et al., 2015 [24] | Rural applicants had a lower ATAR (97.5 ± 0.18 rural vs 99 ± 0.04 urban; *P* <0.001), UMAT 3 percentile scores (63.4 ± 2.5 vs 77.5 ± 0.8; *P* = 0.009 and total UMAT percentile scores (75.8 ± 2 vs 84.6 ± 0.4; *P* = 0.022).  Rural applicants had higher UMAT 2 percentile scores (73.2 ± 2.2) than urban applicants (72.8 ± 0.9; *P* <0.001).  Rural applicants had similar interview scores (26.9 ± 0.6 vs 28 ± 0.2; *P* = 0.065) and UMAT 1 percentile scores (74.5 ± 2.1 vs 79.7 ± 0.7; *P* = 0.865).  For urban origin applicants, UMAT 3 percentile scores were higher for those practicing in an urban (78.1 ± 0.8) compared to rural (69.6 ± 4.1) area (P = 0.046). For urban origin applicants, those working in a rural and urban location had similar (*P* >0.05) ATARs (98.8 ± 0.16 rural vs 98.98 ± 0.04 urban), interview scores (28.3 ± 0.7 vs 28 ± 0.2), UMAT 1 percentile scores (79.9 ± 0.7 vs 77.4 ± 2.7), UMAT 2 percentile scores (71.8 ± 3 vs 72.8 ± 0.9), and total UMAT percentile scores (81.6 ± 1.7 vs 84.8 ± 0.5).  For rural origin applicants, those working in rural and urban locations had similar (*P* >0.05) ATARs (97.36 ± 0.45 vs 97.54 ± 0.2), interview scores (27 ± 1.1 vs 26.9 ± 0.7), UMAT 1 percentile scores (73.6 ± 4.9 vs 74.7 ± 2.4), UMAT 2 percentile scores (76 ± 4.4 vs 72.5 ± 2.5), UMAT 3 percentile scores (63.7 ± 4.6 vs 63.3 ± 2.9), and total UMAT percentile scores (77.4 ± 4.7 vs 75.4 ± 2.2).  In the multivariate regressions, only UMAT 3 scores were associated with practice location. |
| Raghavan et al., 2011 [25] | In the retrospective data, 21.8% of applicants were rural origin and 32.4% of rural and 31.7% of urban applicants were successful.  In the prospective data, there was a 22.4% increase in admission offers, due to a 29-33% increase in the number of eligible rural applicants.  Adjustments to the number of eligible rural applicants was achieved using a weighted aggregate of their ‘rural score’ (Consensus matrix of priorities, identifying three rural attributes). They were given a weighting of ‘no greater than 15%’ which boosts the rural applicant scores by up to 15%. |
| Raghavan et al., 2013 [26] | MMI scores were lower for those who graduate from a rural rather than urban high school (4.4 ± 0.6 vs 4.6 ± 0.7; *P* = 0.003) and had rural connections (4.4 ± 0.7 vs 4.6 ± 0.7; *P* = 0.015).  MMI scores were similar for those who had (4.5 ± 0.7) and had not (4.6 ± 0.7) been employed in a rural area (*P* = 0.268).  MMI scores were similar for those who had (4.5 ± 0.7) and had not (4.5 ± 0.7) completed rural community service (*P* = 0.797).  Applicants from rural compared to urban high schools had lower MCAT writing sample scores (10.2 ± 1.8 vs 10.6 ± 1.7; *P* = 0.0009), MCAT biological science scores (10.5 ± 1.6 vs 10.8 ± 1.7; *P* = 0.023), MCAT physical science scores (9.9 ± 1.9 vs 10.3 ± 2.0; *P* = 0.012), and total MCAT scores (10.1 ± 1.0 vs 10.3 ± 1.1; *P* = 0.0003).  Applicants from rural and urban high schools had similar MCAT reasoning scores (9.6 ± 1.3 vs 9.7 ± 1.4; *P* = 0.696) and GPA (4.0 ± 0.3 vs 4.1 ± 0.2; *P* = 0.170).  In applicants from urban high schools, GPA (rho = 0.093; *P* = 0.003), MCAT (rho = 0.174; *P* <0.0001), MCAT verbal reasoning (rho = 0.175; *P* <0.0001), and MCAT writing sample (rho = 0.250; *P* <0.0001) were significantly correlated with MMI scores.  In applicants from rural high schools, only MCAT writing sample (rho = 0.199; *P* = 0.004) was correlated with MMI scores. |
| Ray, Woolley & Sen Gupta, 2015 [27] | OP scores were progressively higher (worse) for RA 1 (1.9 ± 1.2), RA2 (2.7 ± 2.0), RA3 (3.0 ± 1.8), and RA 4 and 5 applicants (3.6 ± 2.2; *P* <0.001).  Interview scores decreased in applicants from RA 1 (84.5 ± 7.7), RA 2 (82.6 ± 8.3), and RA 3 (81.1 ± 9.4) and RA 4/5 areas (81.6 ± 10.8; *P* <0.001).  OP scores were higher (worse) in students with BMPs (3.3 ± 1.4) compared to CSPs (2.6 ± 1.9; *P* <0.001).  Interview scores were lower in students with BMPs (76.9 ± 7.7) compared to CSPs (84 ± 8.5; *P* <0.001). |
| Schmitz et al., 2020 [28] | 79% of public and 56.1% of private medical schools reported a rurally targeted admissions approach.  88.9% of rurally-located and 67.7% of non-rurally located schools had a rurally targeted admissions approach; however, admissions strategies used were similar regardless of whether the schools were or were not rurally located.  Career exploration/counselling/mentoring was completed in high schools by all rurally located and 81.7% non-rurally located medical schools, in health education centre programs by 83.3% rurally located and 65.3% non-rurally located medical schools, at community/technical colleges by 62.5% rurally located and 62% non-rurally located medical schools, at 4-year universities by 87.5% rurally located and 90.1% non-rurally located medical schools, and at post-baccalaureate programs by 62.5% rurally located and 68.8% non-rurally located medical schools.  Academic enhancement was completed in high schools by 28.6% rurally located and 28.2% non-rurally located medical schools, in health education center programs by 50% rurally located and 22.7% non-rurally located medical schools, at technical colleges by 42.9% rurally located and 20.5% non-rurally located medical schools, at 4-year universities by 57.1% rurally located and 46.8% non-rurally located medical schools, and in post-baccalaureate programs by 42.9% rurally located and 45% non-rurally located medical schools.  Admissions preparation activities were conducted at community/technical colleges by 57.1% rurally located and 33.8% non-rurally located medical schools, at 4-year universities by 87.5% rurally located and 54.4% non-rurally located medical schools, and in post-baccalaureate programs by 62.5% rurally located and 45% non-rurally located medical schools.  Articulation agreements were in place with community/technical colleges for 0 rurally located but 8% non-rurally located medical schools, with 4-year universities by 58.7% rurally located and 39% non-rurally located medical schools (*P* <0.05), and with post-baccalaureate programs by 37.5% rurally located and 18.7% non-rurally located medical schools.  When targeting rural applicants during admissions, 62.5% rurally located and 76% non-rurally located medical schools use secondary application questions, 57.1% rurally located and 35.4% non-rurally located medical schools have targeted financial aid, 12.5% rurally located and 22.4% non-rurally located medical schools have modified MCAT cutoffs, 12.5% rurally located and 21% non-rurally located medical schools have reserved places for rural applicants, 12.5% rurally located and 19.5% non-rurally located medical schools have modified GPA cutoffs, all rurally located and 73.4% non-rurally located medical schools use rural physicians as interviewers, 37.5% rurally located and 38.3% non-rurally located medical schools use preferential scoring during interview screening, 37.5% rurally located and 29.3% non-rurally located medical schools medical schools use preferential scoring when determining admission offers, 0 rurally located and 16.1% non-rurally located medical schools have rural applicants undergo a different admissions process, 0 rurally located and 12.2% non-rurally located medical schools have rural applicants complete separate interviews to other applicants, 0 rurally located and 7.5% non-rurally located medical schools have different interview questions for rural applicants, 66.7% rurally located and 91.1% non-rurally located medical schools involve the dean of admissions in the process, 66.7% rurally located and 72.4% non-rurally located medical schools involve rural physicians in the process, and 66.7% rurally located and 60.9% non-rurally located medical schools involve the director of the rural track or equivalent in the admissions process. |
| Turnbull et al., 2003 [29] | Rural origin students increased from 4-12% to 20-22% per year under the revised admissions processes.  Under the old processes, 58% students had high socioeconomic status, 29% had medium SES, and 13% had low SES and under the new processes, 59% had high SES, 29% had medium SES, and 12% low SES.  Under the old processes, 36% of enrolments were from government schools, 52% from private schools, and 12% catholic schools and under the new processes, 31% of enrolments were from government schools, 57% from private schools, and 12% from catholic schools.  Proportion of students who withdrew from the course decreased from 3.1% to 1.9% after the introduction of the new processes. |
| Upadhyay et al., 2017 [30] | In 2010, 2 (3.33%) students admitted were from backward areas (nine-most remote districts of the mid-western, region), 9 (15%) in 2011, and 6 (10.9%) in 2012.  No students in 2010 and 2012 had completed rural work experience and 1 (1.67%) in the 2011 cohort had completed rural work experience. |
| Wright & Woloschuk, 2008 [31] | 3311 (75.1%) applicants were urban background and of these, 889 (26.8%) were interviewed and 355 (39.9%) were admitted.  866 (19.7%) applicants were from a regional background, with 182 (21%) interviewed and 77 (42.3%) admitted.  230 (5.2%) applicants were from a rural background and of these, 67 (29.1%) were interviewed and 31 (46.3%) were admitted.  A significantly higher proportion of urban and rural applicants were interviewed (*P* <0.05); however, the proportion of rural, urban, and regional applicants admitted was similar (*P* >0.05).  Regional, rural, and urban applicants had similar GPA, MCAT, and reviewer scores (*P* >0.05). |

Note: aOR = adjusted Odds Ratio; OR = Odds Ratio; GPA = Grade Point Average; GAMSAT = Graduate Medical school Admissions test; MMI = Multiple Mini-Interview; SES = Socioeconomic status; MCAT = Medical College Admissions Test; BMP = Bonded Medical Place; RA = Remoteness area; MM = Modified Monash; ATAR = Australian tertiary admissions Rank; UMAT = Undergraduate Medical and Health Sciences Admissions Test; ARIA = Accessibility/Remoteness Index of Australia; AHEC = Area Health education Centre; JCEWT = Joint College Entrance Written Test; NCT = National Centre Test.

**References**

1. Ballejos MP, Oglesbee S, Hettema J, Sapien R. An equivalence study of interview platform: Does videoconference technology impact medical school acceptance rates of different groups? Adv Health Sci Educ. 2018;23:601-10.

2. Beattie J, D'Souza K, Mc Leod J, Versace V. Rural origin students match metropolitan origin students' academic performance once admitted to Bachelor of Medicine Bachelor of Surgery course. Aust J Rural Health. 2019;27:181-2.

3. Curtis E, Wikaire E, Jiang Y, McMillan L, Loto R, Poole P, et al. Examining the predictors of academic outcomes for indigenous Māori, Pacific and rural students admitted into medicine via two equity pathways: A retrospective observational study at the University of Auckland, Aotearoa New Zealand. BMJ Open. 2017;7:e017276.

4. Dallaghan GLB, Spero JC, Byerley JS, Rahangdale L, Fraher EP, Steiner B. Efforts to recruit medical students from rural counties: A model to evaluate recruitment efforts. Cureus J Med Sci. 2021;13: e17464.

5. Evans DV, Jopson AD, Andrilla CHA, Longenecker RL, Patterson DG. Targeted medical school admissions: A strategic process for meeting our social mission. Fam Med. 2020;52:474-82.

6. Fox JL, Batacan R, Saluja S, Pullen C, McGrail M. Experiences of rural and metropolitan background applicants in preparing for and completing a regionally focused multiple mini-interview for admission into a regional medical program. Educ Health. 2023;36:116-22.

7. Gilbert GE, Blue AV, Basco WT. The effect of undergraduate GPA selectivity adjustment on pre-interview ranking of rural medical school applicants. J Rural Health. 2003;19:101-4.

8. Glasser M, Hunsaker M, Sweet K, MacDowell M, Meurer M. A comprehensive medical education program response to rural primary care needs. Acad Med. 2008;83:952-61.

9. Griffin B, Auton J, Duvivier R, Shulruf B, Hu W. Applicants to medical school: If at first they don't succeed, who tries again and are they successful? Adv Health Sci Educ. 2019;24:33-43.

10. Griffin B, Horton GL, Lampe L, Shulruf B, Hu W. The change from UMAT to UCAT for undergraduate medical school applicants: Impact on selection outcomes. Med J Aust. 2021;214:84-9.

11. Hay M, Mercer AM, Lichtwark I, Tran S, Hodgson WC, Aretz HT, et al. Selecting for a sustainable workforce to meet the future healthcare needs of rural communities in Australia. Adv Health Sci Educ. 2017;22:533-51.

12. Hutten-Czapski P, Pitblado R, Rourke J. Who gets into medical school? Comparison of students from rural and urban backgrounds. Can Fam Physician. 2005;51:1240-1.

13. Langer T, Ruiz C, Tsai P, Adams U, Powierza C, Vijay A, et al. Transition to multiple mini interview (MMI) interviewing for medical school admissions. Perspect Med Educ. 2020;9:229-35.

14. Larkins S, Michielsen K, Iputo J, Elsanousi S, Mammen M, Graves L, et al. Impact of selection strategies on representation of underserved populations and intention to practise: International findings. Med Educ. 2015;49(1):60-72.

15. Lin CH, Chen MH, Tsai TC, Huang WJ. Difference in demographics and motivation to study medicine with respect to medical students' channel of admission: A national study. Med Teach. 2021;43:1025-30.

16. Longo DR, Gorman RJ, Ge B. Rural medical school applicants: Do their academic credentials and admission decisions differ from those of nonrural applicants? J Rural Health. 2005;21:346-50.

17. Matsumoto M, Inoue K, Kajii E. Characteristics of medical students with rural origin: Implications for selective admission policies. Health Policy. 2008;87:194-202.

18. Ozeki S, Kasamo S, Inoue H, Matsumoto S. Does regional quota status affect the performance of undergraduate medical students in Japan? A 10-year analysis. Int J Med Educ. 2022;13:307-14.

19. Pang N, Kadir F, Kamu A, Mun HC, Loo JL, Ahmedy F, et al. A pilot project to introduce the multiple mini interview (MMI) at a Borneo medical school: The universiti Malaysia Sabah experience- A cross-sectional study. Ann Med Surg. 2021;71.

20. Puddey IB, Mercer A. Socio-economic predictors of performance in the Undergraduate Medicine and Health Sciences Admission Test (UMAT). BMC Med Educ. 2013;13:155.

21. Puddey IB, Mercer A. Predicting academic outcomes in an Australian graduate entry medical programme. BMC Med Educ. 2014;14:31.

22. Puddey IB, Mercer A, Carr SE, Louden W. Potential influence of selection criteria on the demographic composition of students in an Australian medical school. BMC Med Educ. 2011;11:97.

23. Puddey IB, Mercer A, Playford DE, Pougnault S, Riley GJ. Medical student selection criteria as predictors of intended rural practice following graduation. BMC Med Educ. 2014;14:218.

24. Puddey IB, Mercer A, Playford DE, Riley GJ. Medical student selection criteria and socio-demographic factors as predictors of ultimately working rurally after graduation. BMC Med Educ. 2015;15.

25. Raghavan M, Martin BD, Roberts D, Aoki F, MacKalski BA, Sandham JD. Increasing the enrolment of rural applicants to the faculty of medicine and addressing diversity by using a priority matrix approach to assign values to rural attributes. Rural Remote Health. 2011;11:1646.

26. Raghavan M, Martin BD, Burnett M, Aoki F, Christensen H, Mackalski B, et al. Multiple mini-interview scores of medical school applicants with and without rural attributes. Rural Remote Health. 2013;13:2362.

27. Ray RA, Woolley T, Sen Gupta T. James Cook University's rurally orientated medical school selection process: Quality graduates and positive workforce outcomes. Rural Remote Health. 2015;15:3424.

28. Schmitz DF, Evans DV, Andrilla CHA, Jopson AD, Longenecker RL, Patterson DG. Challenges and best practices for implementing rurally targeted admissions in u.S. medical schools. J Health Care Poor Underserved. 2020;31:320-31.

29. Turnbull D, Buckley P, Robinson JS, Mather G, Leahy C, Marley J. Increasing the evidence base for selection for undergraduate medicine: Four case studies investigating process and interim outcomes. Med Educ. 2003;37:1115-20.

30. Upadhyay SK, Bhandary S, Bhandari DB, Dulal RK, Baral KP, Gongal RN, et al. Admitting deserving medical students from rural and disadvantaged: Patan Academy of Health Sciences' approach. J Nepal Health Res Counc. 2017;15:75-80.

31. Wright B, Woloschuk W. Have rural background students been disadvantaged by the medical school admission process? Med Educ. 2008;42:476-9.
