# Supplementary material for: Medical school admission processes to target rural applicants: an international scoping review and mapping of Australian practices
Source: BMC Med Educ. 2025 May 6;25:659. doi: 10.1186/s12909-025-07234-3 (PMC12057111; doi:10.1186/s12909-025-07234-3)
Supplement: Supplementary file 7 — Supplementary Material 7 [file 12909_2025_7234_MOESM7_ESM.docx]

**Supplementary Table 7.** Qualitative results from studies included in the scoping review

| **Study** | **Key findings** |
| --- | --- |
| Fox et al., 2023 [1] | - When reflecting on the appropriateness of the MMI for selecting students for a rural program, respondents felt it was an important tool for assessing nonacademic qualities and interest in rural work, which were seen as essential for graduates of a rural program. Others felt that performance on the MMI related to access to resources or coaching which “is not accessible to everyone.” - Rural applicants felt the regionally focussed interview questions gave them an advantage in being able to draw from firsthand knowledge and experience aligned with the goals of the medical program. Metropolitan applicants echoed this sentiment and felt that they were disadvantaged by the regional focus of some questions due to insufficient knowledge/ experience. - Most rural applicants felt that any advantage they have in the interview simply counteracts disadvantages they face such as lower quality schooling and educational opportunities. Some metropolitan applicants also felt that they are advantaged in other parts of the admissions process. |
| Henry, Edwards & Crotty, 2009 [2] | All nine medical schools use undergraduate GPA and GAMSAT scores to select for interview and a combination of GPA, GAMSAT, and interview for admissions decisions with one medical also requiring submission of a personal portfolio.  Theme 1, rurality as a factor in selecting for interview:   - All graduate entry schools met the Government requirement of 25% CSPs going to rural background applicants - One school calculates a rurality score and one is developing a rural index. Remaining universities do not quantify applicant’s rurality but one uses a portfolio which includes screening of suitability for rural practice. - Medical schools with rural streams/pathways give preference to rural background applicants. - Universities adjust pre- and post-interview rankings based on applicant’s background to meet rural quotas; however, no schools accept rural applicants with a GPA or GAMSAT below the minimum score published.   Theme 2, Assessment of rurality in selection interviews:   - Six medical schools use a structured interview with two to three panel members and three use the MMI of 8-10 stations for 5-10 minutes each - Panel interviews and MMIs assess similar attributes using a combination of scenarios, case studies, and direct questions, with one also using a mock problem-based learning tutorial - Six medical schools do not directly explore rurality/rural issues during interviews, two schools using MMIs include rural aspects in interview stations, and one medical school which uses a panel interview explores rurality more extensively in the RCS but not other cohorts. One medical school send a rurally-oriented questionnaire (after shortlisting has occurred post-interview) and selection decisions take into account the applicant’s responses.   Theme 3, rural representation on interview panels:   - Five medical schools include rural medical practitioners and/or community members on their interview panels. Participants commented that this can be challenging, particularly in states with larger geographic areas.   Theme 4, rural experience during graduate entry medical courses:   - All medical schools offer a compulsory or elective rural placement in the early years of the graduate entry program ranging from 1 – 8-week placements - In later clinical years, longer placements are offered from 6 weeks to 2.5 years. Longer placements are undertaken by RCS/rural stream students - Four medical schools have rural medicine integrated across the entire course, four conduct full or partial dedicated rural streams, and the last is planning a new course framework that includes rural medicine - Overall, all graduate entry medical schools have short rural placements for all students and greater rural exposure for rural stream students. |
| Larkins et al., 2015 [3] | - Strategies include quotas, selecting on the basis of personal attributes, community involvement in the selection process, and marketing of the school’s social accountability mission. |
| Raghavan et al., 2011 [4] | - Consensus matrix construct of priorities, identified 3 focus attributes: Rural community service (29.1%), rural connections (46.2%), and rural employment (24.7%) |
| Schmitz et al., 2020 [5] | Theme 1, motivations for targeted admissions strategies of key importance:   - Fulfilling the school’s mission was a motivator for many programs - Institutions were often motivated to have rurally targeted admissions processes to train healthcare providers for rural, medically underserved, and primary care, for workforce diversity, and to meet community needs   Theme 2, resources identified as necessary for program success:   - Recruitment/selection of applicants likely to work rurally was resource-intensive - Funding and human resources were reported as necessary and support from institutional leadership was described as very important   Theme 3, challenges identified to program success:   - Recruiting rural applicants was time-intensive and required dedicated personnel - High tuition costs and limited scholarships made it difficult to fill positions with rural applicants - It was challenging during interviews to discern those with genuine intent to practice rurally, particularly for early assurance programs (accepted early in the undergraduate degree)   Theme 4, recommendations for success of targeted admissions strategies:   - Institutional support was identified as critical - Outreach to applicants from rural areas and sufficient resources to do this was advised - Moving away from the emphasis of academic metrics and towards a more holistic selection process was deemed important. |

*Note:* MMI = Multi Mini-Interview; GPA = Grade Point Average; GAMSAT = Graduate Medical School Admissions Test; CSP = Commonwealth Supported Place; RCS = Rural Clinical School; UCAT = University Clinical Aptitude Test.

**References**

1. Fox JL, Batacan R, Saluja S, Pullen C, McGrail M. Experiences of rural and metropolitan background applicants in preparing for and completing a regionally focused multiple mini-interview for admission into a regional medical program. Educ Health. 2023;36:116-22.

2. Henry JA, Edwards BJ, Crotty B. Why do medical graduates choose rural careers? Rural Remote Health. 2009;9:1083.

3. Larkins S, Michielsen K, Iputo J, Elsanousi S, Mammen M, Graves L, et al. Impact of selection strategies on representation of underserved populations and intention to practise: International findings. Med Educ. 2015;49(1):60-72.

4. Raghavan M, Martin BD, Roberts D, Aoki F, MacKalski BA, Sandham JD. Increasing the enrolment of rural applicants to the faculty of medicine and addressing diversity by using a priority matrix approach to assign values to rural attributes. Rural Remote Health. 2011;11:1646.

5. Schmitz DF, Evans DV, Andrilla CHA, Jopson AD, Longenecker RL, Patterson DG. Challenges and best practices for implementing rurally targeted admissions in u.S. medical schools. J Health Care Poor Underserved. 2020;31:320-31.
